# Prevalence, diagnostic methods, and clinical outcomes of wasting/cachexia among pediatric cancer patients in Africa: A protocol for a systematic review and meta-analysis

**DOI:** 10.1371/journal.pone.0349113

**Published:** 2026-05-08

**Authors:** Ivaan Pitua, Daisy Wannyana, Derrick Bary Abila, Felix Bongomin

**Affiliations:** 1 School of Medicine, College of Health Sciences, Makerere University, Kampala, Uganda; 2 Faculty of Health Sciences, Victoria University, Kampala, Uganda; 3 Uganda Child Cancer Foundation, Kampala, Uganda; 4 Uganda Cancer Institute, Kampala, Uganda; 5 Department of Medical Microbiology, Faculty of Health Sciences, Gulu University, Gulu, Uganda; Institute of Public Health from Guanajuato State, MEXICO

## Abstract

**Introduction:**

Pediatric cancer is an emerging public health priority in Africa, where survival rates remain critically low compared to high-income regions. Malnutrition; specifically wasting and cachexia; is the most prevalent, yet modifiable comorbidity that compromises treatment tolerance and increases mortality. Recent primary studies from 2025 indicate a significant discrepancy between wasting diagnosed via clinical assessment versus anthropometrically defined wasting, suggesting a “hidden burden” of malnutrition in African oncology wards. However, no comprehensive synthesis of data exists regarding the prevalence of wasting across the continent using modern assessment standards, nor its specific impact on clinical outcomes in the current treatment era.

**Methods and analysis:**

We will conduct a systematic review and meta-analysis of observational studies (cross-sectional, cohort, and case-control) published from January 1, 2000, to the present. Data sources will include PubMed/MEDLINE, EMBASE, Web of Science and CINAHL. We will include studies involving children and adolescents (0–19 years) diagnosed with malignancies in African healthcare settings. Two independent reviewers will screen studies, extract data, and assess risk of bias using Covidence systematic review software. The risk of bias will be assessed using the Joanna Briggs Institute (JBI) critical appraisal tools. The primary outcome will be the pooled prevalence of wasting/cachexia. Secondary outcomes will include diagnostic accuracy of assessment methods (such as Mid-Upper Arm Circumference [MUAC] vs. Weight-for-Height vs. clinical assessment) and associations with adverse clinical events (neutropenia, sepsis, treatment abandonment, and mortality). A random-effects meta-analysis will be performed using R software. Heterogeneity will be assessed using the I^2^ statistic and explored via subgroup analyses (region, tumor type, and assessment tool).

**Ethics and dissemination:**

Ethical approval is not required as this study relies on secondary data. Findings will be disseminated through a peer-reviewed publication and conference presentations to inform nutritional guidelines for pediatric oncology in resource-limited settings.

**PROSPERO Registration Number:**

**Registration Number:** CRD420251237859

## Introduction

Childhood cancer represents a growing global health challenge, with the burden disproportionately shifting toward Low- and Middle-Income Countries (LMICs), particularly in Africa. While High-Income Countries have achieved survival rates exceeding 80%, survival in sub-Saharan Africa remains starkly lower, often estimated below 30% [1,2]. The WHO Global Initiative for Childhood Cancer (GICC) aims to achieve a minimum 60% survival rate for children with cancer in all countries by 2030, with equity as a core principle [2]. Achieving quality multimodal therapy and sustainable follow-up care is fundamentally compromised by malnutrition. Nutritional depletion reduces chemotherapy tolerability, increases treatment-related toxicity, and prolongs hospitalization, all of which undermine the GICC’s equity goals. Yet structural barriers, including the absence of standardized nutritional screening protocols and dedicated dietitian services, persist across African oncology settings [2,3]. Beyond the lack of access to cytotoxic drugs and radiotherapy, malnutrition remains a critical, pervasive, and modifiable prognostic factor that exacerbates mortality [3–7]. Recent systematic reviews indicate that while global attention focuses on therapeutics, the basic nutritional platform required to tolerate these therapies is often missing in African settings [1].

In the African context, pediatric oncology patients face a “double burden” of malnutrition. Children often present with pre-existing undernutrition due to widespread food insecurity before the malignancy is diagnosed [7]. Wasting, defined as low weight-for-height or low Mid-Upper Arm Circumference (MUAC), is a critical indicator of acute malnutrition and immediate mortality risk.

The biological interaction between malnutrition and cancer is complex. The presence of a tumor induces a hypermetabolic state driven by inflammatory cytokines, promoting muscle proteolysis and lipolysis—a syndrome known as cancer cachexia [8]. When this cachectic drive is superimposed on a child with limited nutritional reserves, the physiological impact is catastrophic. Malnutrition leads to immune suppression, altered drug pharmacokinetics, and reduced gut mucosal integrity [3,9]. Consequently, malnourished children experience higher rates of severe neutropenia, sepsis, and treatment toxicity, which are leading causes of preventable mortality in African oncology wards [4,10].

A critical barrier to addressing this crisis is the inconsistency of assessment tools. In many African hospitals, nutritional assessment relies on weight-based metrics or subjective clinical judgment. However, emerging evidence suggests these metrics are unreliable in oncology; large solid tumors (such as Wilms tumor) or organomegaly can artificially inflate body weight, masking severe somatic protein depletion [11]. Similarly, clinical presentations like edema can limit the utilization of both MUAC and Weight based anthropometries.

Studies published in 2025 from Uganda have highlighted significant diagnostic gaps. Nyeko et al. (2025) found that relying on “visible wasting” (clinical judgment) had a sensitivity of only 43.9% compared to anthropometry, missing 80.6% of children with moderate wasting [10]. Similarly, Wannyana et al. (2025) identified a wasting prevalence of 27.4% using MUAC, identifying specific vulnerabilities in older children (>5 years) and those with gastrointestinal tumors [12]. This data corroborates recent findings from Tanzania and South Africa, where malnutrition rates vary significantly depending on the tool used, with MUAC often revealing a higher burden than BMI in patients with solid tumors [6,13].

Despite these insights, clinical practice varies widely. Many centers lack dedicated dietitians, and national malnutrition protocols often neglect older children and adolescents, focusing primarily on under-fives [5]. While individual studies from Uganda [10,12], Malawi [5], Ghana [14], and South Africa [6] have reported high prevalence rates of wasting, there is no comprehensive, pan-African synthesis of data. Understanding the true magnitude of wasting across the continent is essential for advocacy. If wasting is consistently underdiagnosed by current methods, as suggested by Nyeko and colleagues [10] then mortality attributed to “disease progression” may actually be driven by malnutrition-induced toxicity [15]. Furthermore, although a study conducted in Nigeria assessed the use of accuracy of WFH and MUAC, there is a dearth of data regarding a comprehensive analysis of diagnostic accuracy of assessment methods (MUAC vs. Weight-for-Height vs. clinical assessment) and associations with adverse clinical events (neutropenia, sepsis, treatment abandonment, and mortality) 20. This review aims to synthesize the prevalence of wasting and cachexia among pediatric cancer patients in Africa, characterize the diagnostic performance of assessment tools used in clinical and field settings, and quantify the association between nutritional status and adverse clinical outcomes including mortality, sepsis, neutropenia, and treatment abandonment.[2]

## Methods

This protocol is drafted in accordance with the Preferred Reporting Items for Systematic Review and Meta-Analysis Protocols (PRISMA-P) guidelines [16]. Full cheklist in S2 File.

### Eligibility criteria

#### Study designs.

- Inclusion: Observational studies, including cross-sectional, prospective, and retrospective cohorts, and case-control studies that report prevalence data or associations with clinical outcomes.
- Exclusion: Case reports (<10 patients), conference abstracts, reviews, editorials, qualitative studies, and animal studies. General conference abstracts are excluded.
- Exception for Grey Literature: Proceedings from the International Society of Paediatric Oncology (SIOP) will be included only if they provide sufficient quantitative data (sample size, clear definition of wasting, and prevalence rates). If an abstract lacks essential data, authors will be contacted. If no response is received, the study will be excluded from the quantitative synthesis.

#### Participants.

- Inclusion: Children and adolescents (aged 0–19 years) with a confirmed diagnosis of any malignancy (hematological or solid tumors).
- Setting: Healthcare facilities located within the African continent (defined by African Union member states).

#### Exposure/Condition.

- Wasting, cachexia, or acute malnutrition defined by any recognized standard:
  - Anthropometry: Z-scores < −2 SD (moderate) or <−3 SD (severe) for Weight-for-Height/Length (WFH/L), BMI-for-age, or Mid-Upper Arm Circumference (MUAC) (severe acute malnutrition if MUAC is less than 11.5 cm (≤−3 Z-scores) and moderate acute malnutrition if MUAC is 11.5–12.4 cm (≤−2 Z-scores) for children ages 6−59 months, for older children, MUAC readings corresponding with Z-scores: 0, −2 to −2.9 and ≤−3 will be interpreted as no wasting, moderate wasting and severe wasting respectively 19.
- Clinical Assessment: Subjective Global Assessment (SGA) or documented “visible wasting.”

**Comparators (for outcome analysis):**

- Children with cancer who are well-nourished or have normal anthropometric status.

### Outcomes

Primary outcome will be pooled prevalence of wasting/cachexia (overall and stratified by severity: moderate vs. severe) among pediatric cancer patients in Africa while secondary outcomes will include; Diagnostic performance (sensitivity, specificity) of clinical assessment versus objective anthropometry (MUAC, Weight-for-Height, BMI-for-age); Association between wasting and adverse clinical outcomes expressed as Odds Ratios or Relative Risks for: all-cause mortality, Grade 3/4 neutropenia, sepsis, and treatment abandonment; and Variation in wasting prevalence by region, cancer type, age group, and assessment tool.

### Information sources

We will search the following electronic databases:

1. PubMed/MEDLINE
2. EMBASE
3. CINAHL (Cumulative Index to Nursing and Allied Health Literature)
4. Africa Science Online
5. Web of Science
6. Grey Literature: To reduce publication bias, we will specifically search the SIOP (International Society of Paediatric Oncology) Africa conference proceedings and abstract books.

### Search strategy

A comprehensive search strategy will be developed using Medical Subject Headings (MeSH) and free-text keywords. The strategy will combine terms for the population (Pediatrics), condition (Cancer), outcome (Malnutrition/Wasting), and setting (Africa).

Example PubMed Search String (full example database search strings from different sources in S1 File):

(“Child”[Mesh] OR “Adolescent”[Mesh] OR pediatric* OR paediatric* OR infant*) AND (“Neoplasms”[Mesh] OR cancer* OR leukemia OR lymphoma OR tumor* OR malignancy) AND (“Wasting Syndrome”[Mesh] OR “Cachexia”[Mesh] OR “Malnutrition”[Mesh] OR “Thinness” OR “Mid Upper Arm Circumference” OR “MUAC” OR “Body Mass Index” OR “Weight loss”) AND (“Africa”[Mesh] OR “Sub-Saharan Africa” OR “Uganda” OR “Kenya” OR “Nigeria” OR “South Africa” [AND other individual country names])

### Study selection

Search results will be imported into Covidence systematic review software (Veritas Health Innovation, Melbourne, Australia) for deduplication and screening. Two independent reviewers will screen titles and abstracts for relevance. Full-text articles of potentially eligible studies will be retrieved and assessed against inclusion criteria. Disagreements will be resolved by consensus or consultation with a third reviewer. A PRISMA flow diagram will document the selection process.

### Data extraction

Data will be extracted into a standardized Microsoft Excel form piloted on five random studies.

Data items to be collected include:

- Study characteristics: Author, year, country, region, study design, sample size, data collection period.
- Participant details: Mean/median age or age groups, sex distribution, cancer types (proportion solid vs. liquid).
- Assessment details: Method of nutritional assessment (e.g., WHO growth standards, CDC charts), specific tool used (MUAC, WFH, BMI), and cut-offs for definition as well as that for clinical assessment (clinical assessment).
- Prevalence data: Total number of participants, number wasted (moderate vs. severe), prevalence of stunting (if reported alongside wasting).
- Outcome data: Number of events (deaths, sepsis episodes) in wasted vs. non-wasted groups; adjusted and unadjusted effect estimates.

### Quality assessment (Risk of Bias)

Two reviewers will independently assess study quality:

- **For Prevalence Data:** The Joanna Briggs Institute (JBI) Checklist for Prevalence Studies will be used (assessing sampling frame, sample size, and standard measurement).
- **For Cohort/Outcome Data:** The Newcastle-Ottawa Scale (NOS) will be adapted to assess selection, comparability, and outcome ascertainment.

### Data synthesis and analysis

#### Prevalence analysis.

We will perform a meta-analysis using R Statistical Software (using meta and metafor packages). The pooled prevalence of wasting will be calculated using the Freeman-Tukey double arcsine transformation to stabilize variances. Due to anticipated heterogeneity in healthcare settings across Africa, a random-effects model (DerSimonian-Laird method) will be used.

### Measures of association

For clinical outcomes (mortality, toxicity), we will pool Odds Ratios (OR) or Relative Risks (RR) using the inverse variance method.

### Heterogeneity and subgroup analysis

Heterogeneity will be assessed using the I^2^ statistic and Cochran’s Q test. Values of I^2^ > 50% will imply substantial heterogeneity. We will conduct subgroup analyses to explore variations based on:

1. **Assessment Method:** Studies using MUAC vs. Weight-based measures vs. Clinical assessment.
2. **Region:** East, West, North, South, and Central Africa.
3. **Cancer Classification:** Solid tumors (where weight may be confounded by tumor mass) vs. Hematological malignancies.
4. **Age Group:** Children (<5 years) vs. Older Children/Adolescents (≥5 years).

### Sensitivity analysis

We will perform sensitivity analyses by excluding studies with high risk of bias and “outlier” studies to assess the robustness of the results.

### Publication bias

If 10 or more studies are included, we will assess publication bias using funnel plots and Egger’s regression test.

### Assessment of certainty of evidence

Two independent reviewers will assess the certainty of the evidence for the secondary outcomes (associations between wasting and adverse clinical events) using the Grading of Recommendations Assessment, Development and Evaluation (GRADE) approach [17]. We will evaluate the evidence based on five domains: risk of bias, inconsistency, indirectness, imprecision, and publication bias. The overall certainty of evidence will be graded as high, moderate, low, or very low. We will use the GRADEpro GDT software (McMaster University, ON, Canada) to create Summary of Findings (SoF) tables. For prevalence estimates, we will summarize the quality of evidence based on the risk of bias assessment using the JBI critical appraisal tool.

### Data management and sharing

Upon completion of the systematic review, all study-level extracted data underlying reported findings will be deposited in a public open-access repository (Open Science Framework, https://osf.io) and made freely available without restriction.

### Study status and timeline

This systematic review is currently in the protocol stage. Preliminary database searches have been piloted to refine the search strategy, but formal search, screening and data extraction have not yet commenced. The anticipated timeline for the study is as follows:

Database Searching and Screening: December 1, 2025 – December 14, 2025.

Data Extraction and Quality Assessment: December 15, 2025 – December 31, 2025.

Data Synthesis and Analysis: January 1, 2026 – January 15, 2026.

Expected Completion and Manuscript Submission: Late January 2026

## Discussion

### Interpretation of anticipated findings

The results from this review are anticipated to confirm that wasting remains a highly prevalent comorbidity, potentially affecting 30–50% of newly diagnosed patients [1], and to provide the evidence base necessary to guide nutritional policy in African pediatric oncology settings.

A central theme of our discussion will be the critical discrepancy between assessment methods. Evidence from Nyeko et al. (2025) suggests that clinical practice in resource-limited settings often relies on “visual” diagnosis, which has poor sensitivity [10]. We expect our meta-analysis to reveal higher pooled prevalence rates in studies utilizing objective anthropometry (specifically MUAC) compared to those relying on clinical assessment or weight-for-height indices [5,10]. This finding would reveal a “hidden burden” of malnutrition, children who are physiologically compromised but visibly “normal,” particularly those with large abdominal tumors where tumor mass confounds weight-based assessments [6].

### Clinical implications: The malnutrition-toxicity cycle

The relationship between wasting and adverse clinical outcomes is expected to be bidirectional. Malnourished children have reduced physiological reserves and altered pharmacokinetics, leading to higher rates of Grade 3/4 neutropenia and sepsis, as noted in recent Ugandan and Tanzanian cohorts [10,13]. This toxicity often necessitates chemotherapy dose reductions, which compromises event-free survival. Furthermore, the immunosuppression associated with wasting makes these children uniquely vulnerable to hospital-acquired infections [4]. Our review will quantify this risk, arguing that nutritional rehabilitation is a vital component of “toxicity management” and infection control [9].

### Age-specific vulnerabilities

We also intend to highlight the neglected demographic of older children and adolescents. Wannyana and colleagues found a higher prevalence of wasting in children aged over 5 years compared to younger children [12]. This is a crucial finding for policy, as most nutritional programs in Africa (such as ready-to-use therapeutic food distribution) are often restricted to children under 5 years of age [18]. Adolescents with cancer may have rapid growth requirements that, when combined with cancer cachexia, lead to profound muscle wasting (sarcopenia) that goes unrecognized by standard pediatric growth charts [15,19].

### Policy and research recommendations

Should our findings demonstrate superior diagnostic performance for MUAC, this information could be used to inform evidence-based recommendations for the integration of objective nutritional screening tools into routine pediatric oncology triage protocols across African healthcare settings [11]. Furthermore, we will advocate for the adoption of nutritional guidelines adapted for local food availability [7].

### Strengths and limitations

This review’s strength lies in its pan-African scope and rigorous stratification by diagnostic tool using only the most current data. However, limitations will likely include the heterogeneity of included studies, many of which may be single-center, retrospective audits with varying definitions of malnutrition.

## Conclusion

Wasting and cachexia represent a silent emergency within the pediatric oncology landscape of Africa. While advancements in chemotherapy and surgery are critical, their efficacy is fundamentally undermined when administered to a physiologically depleted child. This systematic review aims to provide the definitive epidemiological evidence required to shift malnutrition from a “peripheral concern” to a central pillar of pediatric cancer care in Africa.

By synthesizing data from 2020 to 2025, we aim to demonstrate that the burden of wasting is not only high but often invisible to standard clinical assessment methods. The anticipated findings will likely challenge the reliance on weight-based metrics and emphasize the urgency of implementing low-cost, high-sensitivity tools like MUAC in routine oncological triage.

## Supporting information

S1 File Database search strings. Full search strategies for PubMed/MEDLINE, EMBASE, CINAHL, Web of Science, and Africa Science Online.
(DOCX)

S2 File PRISMA-P Checklist. Preferred Reporting Items for Systematic Review and Meta-Analysis Protocols checklist with page-number citations.
(DOCX)
