# Supplementary material for: Prevalence, diagnostic methods, and clinical outcomes of wasting/cachexia among pediatric cancer patients in Africa: A protocol for a systematic review and meta-analysis
Source: PLoS One. 2026 May 8;21(5):e0349113. doi: 10.1371/journal.pone.0349113 (PMC13155595; doi:10.1371/journal.pone.0349113)
Supplement: S1 File — Full search strategies for PubMed/MEDLINE, EMBASE, CINAHL, Web of Science, and Africa Science Online. (DOCX) [file pone.0349113.s001.docx]

**S1 File**

**Search strings**

**PubMed/Medline**

| **No** | **Search strategy** | **Description** |
| --- | --- | --- |
| #1 | ("Infant"[Mesh] OR "Child"[Mesh] OR "Adolescent"[Mesh] OR infant[tiab] OR infants[tiab] OR child[tiab] OR children[tiab] OR childhood[tiab] OR adolescent[tiab] OR adolescents[tiab] OR teen[tiab] OR teens[tiab] OR teenage*[tiab] OR youth[tiab] OR youths[tiab] OR pediatr*[tiab] OR paediatr*[tiab]) | Children and adolescents (0-19 years) |
| #2 | Search strategy for cancer (see below) | Cancer |
| #3 | ("Wasting Syndrome"[Mesh] OR "Cachexia"[Mesh] OR "Thinness"[Mesh] OR "Protein-Energy Malnutrition"[Mesh] OR wasting[tiab] OR wasted[tiab] OR "weight loss"[tiab] OR "weight-loss"[tiab] OR underweight[tiab] OR under-weight[tiab] OR undernourish*[tiab] OR malnourish*[tiab] OR "acute malnutrition"[tiab] OR "severe acute malnutrition"[tiab] OR "moderate acute malnutrition"[tiab] OR cachexia[tiab] OR cachectic[tiab] OR emaciat*[tiab] OR "low body mass index"[tiab] OR "low BMI"[tiab] OR "low weight-for-height"[tiab] OR "mid upper arm circumference"[tiab] OR MUAC[tiab]) | Cachexia, malnutrition, and synonyms |
| #4 | ("Africa"[Mesh] OR "Africa South of the Sahara"[Mesh] OR africa[tiab] OR african[tiab] OR "sub-Saharan Africa"[tiab] OR "sub Saharan Africa"[tiab] OR "East Africa"[tiab] OR "West Africa"[tiab] OR "Central Africa"[tiab] OR "Southern Africa"[tiab] OR "North Africa"[tiab] OR Algeria[tiab] OR Angola[tiab] OR Benin[tiab] OR Botswana[tiab] OR "Burkina Faso"[tiab] OR Burundi[tiab] OR "Cabo Verde"[tiab] OR "Cape Verde"[tiab] OR Cameroon[tiab] OR "Central African Republic"[tiab] OR Chad[tiab] OR Comoros[tiab] OR "Democratic Republic of the Congo"[tiab] OR "Congo, Democratic Republic"[tiab] OR "DR Congo"[tiab] OR DRC[tiab] OR "Republic of the Congo"[tiab] OR "Congo-Brazzaville"[tiab] OR Congo[tiab] OR "Cote d'Ivoire"[tiab] OR "Côte d'Ivoire"[tiab] OR "Ivory Coast"[tiab] OR Djibouti[tiab] OR Egypt[tiab] OR "Equatorial Guinea"[tiab] OR Eritrea[tiab] OR Eswatini[tiab] OR Swaziland[tiab] OR Ethiopia[tiab] OR Gabon[tiab] OR Gambia[tiab] OR "The Gambia"[tiab] OR Ghana[tiab] OR Guinea[tiab] OR "Guinea-Bissau"[tiab] OR Kenya[tiab] OR Lesotho[tiab] OR Liberia[tiab] OR Libya[tiab] OR Madagascar[tiab] OR Malawi[tiab] OR Mali[tiab] OR Mauritania[tiab] OR Mauritius[tiab] OR Morocco[tiab] OR Mozambique[tiab] OR Namibia[tiab] OR Niger[tiab] OR Nigeria[tiab] OR Rwanda[tiab] OR "Sahrawi Arab Democratic Republic"[tiab] OR "Western Sahara"[tiab] OR "Sao Tome and Principe"[tiab] OR "São Tomé and Príncipe"[tiab] OR Senegal[tiab] OR Seychelles[tiab] OR "Sierra Leone"[tiab] OR Somalia[tiab] OR "South Africa"[tiab] OR "South Sudan"[tiab] OR Sudan[tiab] OR Tanzania[tiab] OR "United Republic of Tanzania"[tiab] OR Togo[tiab] OR Tunisia[tiab] OR Uganda[tiab] OR Zambia[tiab] OR Zimbabwe[tiab]) | Studies conducted in Africa |
| #5 | #1 AND #2 | Childhood cancer |
| #6 | #3 AND #5 | Cachexia in childhood cancer |
| #7 | #4 AND #6 | Cachexia in childhood cancer in Africa |

**Web of Science Core Collection**

| **No** | **Search strategy** | **Description** |
| --- | --- | --- |
| #1 | TS=(("Infant" OR "Child" OR "Adolescent" OR infant OR infants OR child OR children OR childhood OR adolescent OR adolescents OR teen OR teens OR teenage* OR youth OR youths OR pediatr* OR paediatr*)) | Children and adolescents (0-19 years) |
| #2 | Search strategy for cancer (see below) | Cancer |
| #3 | TS=(("Wasting Syndrome" OR "Cachexia" OR "Thinness" OR "Protein-Energy Malnutrition" OR wasting OR wasted OR "weight loss" OR "weight-loss" OR underweight OR under-weight OR undernourish* OR malnourish* OR "acute malnutrition" OR "severe acute malnutrition" OR "moderate acute malnutrition" OR cachexia OR cachectic OR emaciat* OR "low body mass index" OR "low BMI" OR "low weight-for-height" OR "mid upper arm circumference" OR MUAC)) | Cachexia, malnutrition, and synonyms |
| #4 | TS=(("Africa" OR "Africa South of the Sahara" OR africa OR african OR "sub-Saharan Africa" OR "sub Saharan Africa" OR "East Africa" OR "West Africa" OR "Central Africa" OR "Southern Africa" OR "North Africa" OR Algeria OR Angola OR Benin OR Botswana OR "Burkina Faso" OR Burundi OR "Cabo Verde" OR "Cape Verde" OR Cameroon OR "Central African Republic" OR Chad OR Comoros OR "Democratic Republic of the Congo" OR "Congo, Democratic Republic" OR "DR Congo" OR DRC OR "Republic of the Congo" OR "Congo-Brazzaville" OR Congo OR "Cote d'Ivoire" OR "Côte d'Ivoire" OR "Ivory Coast" OR Djibouti OR Egypt OR "Equatorial Guinea" OR Eritrea OR Eswatini OR Swaziland OR Ethiopia OR Gabon OR Gambia OR "The Gambia" OR Ghana OR Guinea OR "Guinea-Bissau" OR Kenya OR Lesotho OR Liberia OR Libya OR Madagascar OR Malawi OR Mali OR Mauritania OR Mauritius OR Morocco OR Mozambique OR Namibia OR Niger OR Nigeria OR Rwanda OR "Sahrawi Arab Democratic Republic" OR "Western Sahara" OR "Sao Tome AND Principe" OR "São Tomé AND Príncipe" OR Senegal OR Seychelles OR "Sierra Leone" OR Somalia OR "South Africa" OR "South Sudan" OR Sudan OR Tanzania OR "United Republic of Tanzania" OR Togo OR Tunisia OR Uganda OR Zambia OR Zimbabwe)) | Studies conducted in Africa |
| #5 | #1 AND #2 | Childhood cancer |
| #6 | #3 AND #5 | Cachexia in childhood cancer |
| #7 | #4 AND #6 | Cachexia in childhood cancer in Africa |

**EMBASE**

In Embase, we shall search databases of the following:

- Embase
- CINAHL (Cumulative Index to Nursing and Allied Health Literature)
- APA PsycINFO
- ProQuest Dissertations & Theses Global (PQDT)

| **No** | **Search strategy** | **Description** |
| --- | --- | --- |
| #1 | ("Infant" OR "Child" OR "Adolescent" OR infant OR infants OR child OR children OR childhood OR adolescent OR adolescents OR teen OR teens OR teenage* OR youth OR youths OR pediatr* OR paediatr*) | Children and adolescents (0-19 years) |
| #2 | Search strategy for cancer | Cancer |
| #3 | ("Wasting Syndrome" OR "Cachexia" OR "Thinness" OR "Protein-Energy Malnutrition" OR wasting OR wasted OR "weight loss" OR "weight-loss" OR underweight OR under-weight OR undernourish* OR malnourish* OR "acute malnutrition" OR "severe acute malnutrition" OR "moderate acute malnutrition" OR cachexia OR cachectic OR emaciat* OR "low body mass index" OR "low BMI" OR "low weight-for-height" OR "mid upper arm circumference" OR MUAC) | Cachexia, malnutrition, and synonyms |
| #4 | ("Africa" OR "Africa South of the Sahara" OR africa OR african OR "sub-Saharan Africa" OR "sub Saharan Africa" OR "East Africa" OR "West Africa" OR "Central Africa" OR "Southern Africa" OR "North Africa" OR Algeria OR Angola OR Benin OR Botswana OR "Burkina Faso" OR Burundi OR "Cabo Verde" OR "Cape Verde" OR Cameroon OR "Central African Republic" OR Chad OR Comoros OR "Democratic Republic of the Congo" OR "Congo, Democratic Republic" OR "DR Congo" OR DRC OR "Republic of the Congo" OR "Congo-Brazzaville" OR Congo OR "Cote d'Ivoire" OR "Côte d'Ivoire" OR "Ivory Coast" OR Djibouti OR Egypt OR "Equatorial Guinea" OR Eritrea OR Eswatini OR Swaziland OR Ethiopia OR Gabon OR Gambia OR "The Gambia" OR Ghana OR Guinea OR "Guinea-Bissau" OR Kenya OR Lesotho OR Liberia OR Libya OR Madagascar OR Malawi OR Mali OR Mauritania OR Mauritius OR Morocco OR Mozambique OR Namibia OR Niger OR Nigeria OR Rwanda OR "Sahrawi Arab Democratic Republic" OR "Western Sahara" OR "Sao Tome AND Principe" OR "São Tomé AND Príncipe" OR Senegal OR Seychelles OR "Sierra Leone" OR Somalia OR "South Africa" OR "South Sudan" OR Sudan OR Tanzania OR "United Republic of Tanzania" OR Togo OR Tunisia OR Uganda OR Zambia OR Zimbabwe) | Studies conducted in Africa |
| #5 | #1 AND #2 | Childhood cancer |
| #6 | #3 AND #5 | Cachexia in childhood cancer |
| #7 | #4 AND #6 | Cachexia in childhood cancer in Africa |

**Pubmed Search strategy for cancer –**

[**https://www.nlm.nih.gov/bsd/pubmed_subsets/cancer_strategy.html**](https://www.nlm.nih.gov/bsd/pubmed_subsets/cancer_strategy.html)

(neoplasms OR American Cancer Society OR angiogenesis inducing agents OR antibodies, neoplasm OR antigens, neoplasm OR antineoplastic agents OR antineoplastic protocols OR biomarkers, tumor OR biopsy [mh] OR biopsy [tw] OR bone marrow purging OR bone marrow transplantation OR cancer care facilities OR cancer vaccines OR carcinogenicity tests OR carcinogens OR chemoembolization, therapeutic OR clonal evolution [mh] OR clonal evolution [tw] OR colonography, computed tomographic OR colonoscopy OR colposcopy OR combined modality therapy OR cryosurgery OR cytapheresis OR dna, neoplasm OR drug resistance, neoplasm OR drug screening assays, antitumor OR early detection of cancer OR gene expression regulation, neoplastic OR genes, neoplasm OR graft vs tumor effect OR hematopoietic stem cell transplantation OR hematopoietic stem cell mobilization OR immunotherapy, adoptive OR leukostasis OR lymph node excision OR lymphocytes, tumor-infiltrating OR mammography OR mastectomy OR medical oncology OR metastasectomy OR mohs surgery OR myelodysplastic-myeloproliferative diseases OR neoplasm grading OR neoplasm proteins OR neoplasm staging OR neoplasm transplantation OR neoplastic processes OR neoplastic stem cells OR oncogene fusion OR oncogenic viruses OR oncology nursing OR oncology service, hospital OR oncolytic viruses OR papanicolaou test [mh] OR papillomavirus vaccines OR peripheral blood stem cell transplantation OR polyomavirus OR radiotherapy OR radiotherapy planning, computer assisted OR rna, neoplasm OR second-look surgery OR SEER program OR stem cell transplantation [mh:noexp] OR transplantation conditioning OR tumor cells, cultured OR tumor escape OR tumor lysis syndrome OR tumor necrosis factors OR receptors, tumor necrosis factor OR tumor necrosis factor receptor-associated peptides and proteins OR ultrasonography, mammary OR AACR OR AJCC [tw] OR (ASCO NOT fungi) OR IARC OR "National Cancer Institute (U.S.)" [mh] OR UICC OR aCML [tw] OR AGCUS [tw] OR AILD [tw] OR AML [tw] OR ANLL [tw] OR ASCUS [tw] OR ATLL [tw] OR BRCA [tw] OR BRCA1 [tw] OR BRCA2 [tw] OR CIN [tw] OR CLL [tw] OR CMML [tw] OR CMPD [tw] OR ECCL [tw] OR EGIST [tw] OR FMTC [tw] OR GLNH [tw] OR HNPCC [tw] OR HNSCC [tw] OR HPV [tw] OR HSIL [tw] OR ICD O [tw] OR JCML [tw] OR JMML [tw] OR LGLL [tw] OR MGUS [tw] OR MLH1[tw] OR MPD [tw] OR MSH2[tw] OR NSCLC [tw] OR RAEB [tw] OR RCMD [tw] OR SCLC [tw] OR VOD [tw] OR 5q syndrome [tw] OR BCR ABL [tw] OR c erbB 2 [tw] OR c erbB2 [tw] OR carney complex [tw] OR cone biopsy [tw] OR denys drash [tw] OR essential thrombocythemia [tw] OR estrogen receptor negative [tw] OR estrogen receptor positive [tw] OR li fraumeni [tw] OR meigs syndrome [tw] OR molar pregnancy [tw] OR mycosis fungoides [tw] OR peutz jeghers [tw] OR sentinel lymph node [tw] OR sezary syndrome [tw] OR struma ovarii [tw] OR sturge weber [tw] OR zollinger ellison [tw] OR (aberrant [tw] AND crypt [tw] AND foci [tw]) OR ((anti-n-methyl-d-aspartate [tw] OR anti-nmda) AND encephalitis [tw]) OR (barrett [tw] AND esophagus [tw]) OR (gestational [tw] AND trophoblastic [tw]) OR (microsatellite [tw] AND instability [tw]) OR (paget [tw] AND (breast [tw] OR nipple [tw])) OR (polycythemia [tw] AND vera [tw]) OR (radiation [tw] AND therapy [tw]) OR (WAGR [tw] AND syndrome [tw]) OR (pap [tw] AND (smear [tw] OR smears [tw])) OR cervical smear [tw] OR cervical smears [tw] OR pap test [tw] OR pap tests [tw] OR (PSA [tw] AND prostate) OR PSA test [tw] OR PSA testing [tw] OR (prostate [tw] AND specific [tw] AND antigen [tw]) OR acanthoma [tw] OR acanthomas [tw] OR acrochordon [tw] OR acrochordons [tw] OR acrospiroma [tw] OR acrospiromas [tw] OR adamantinoma [tw] OR adamantinomas [tw] OR adenoacanthoma [tw] OR adenoacanthomas [tw] OR adenoameloblastoma [tw] OR adenoameloblastomas [tw] OR adenocanthoma [tw] OR adenocanthomas [tw] OR adenocarcinoma [tw] OR adenocarcinomas [tw] OR adenofibroma [tw] OR adenofibromas [tw] OR adenolipoma [tw] OR adenolipomas [tw] OR adenolymphoma [tw] OR adenolymphomas [tw] OR adenoma [tw] OR adenomas [tw] OR adenomatosis [tw] OR adenomatous [tw] OR adenomyoepithelioma [tw] OR adenomyoepitheliomas [tw] OR adenomyoma [tw] OR adenomyomas [tw] OR adenosarcoma [tw] OR adenosarcomas [tw] OR adenosis [tw] OR aesthesioneuroblastoma [tw] OR aesthesioneuroblastomas [tw] OR ameloblastoma [tw] OR ameloblastomas [tw] OR amyloidoses [tw] OR amyloidosis [tw] OR anaplasia [tw] OR androblastoma [tw] OR androblastomas [tw] OR angioblastoma [tw] OR angioblastomas [tw] OR angioendothelioma [tw] OR angioendotheliomas [tw] OR angioendotheliomatosis [tw] OR angiofibroma [tw] OR angiofibromas [tw] OR angiofibrosarcoma [tw] OR angiogenesis factor [tw] OR angiokeratoma [tw] OR angiokeratomas [tw] OR angioleiomyoma [tw] OR angioleiomyomas [tw] OR angiolipoma [tw] OR angiolipomas [tw] OR angioma [tw] OR angiomas [tw] OR angiomatosis [tw] OR angiomyolipoma [tw] OR angiomyolipomas [tw] OR angiomyoma [tw] OR angiomyomas [tw] OR angiomyxoma [tw] OR angiomyxomas [tw] OR angioreticuloma [tw] OR angioreticulomas [tw] OR angiosarcoma [tw] OR angiosarcomas [tw] OR anticancer [tw] OR anticarcinogenesis [tw] OR anticarcinogenic [tw] OR antimutagenesis [tw] OR antineoplastic [tw] OR antioncogene [tw] OR antioncogenes [tw] OR antitumor [tw] OR antitumors [tw] OR antitumour [tw] OR antitumours [tw] OR apudoma [tw] OR apudomas [tw] OR argentaffinoma [tw] OR argentaffinomas [tw] OR arrhenoblastoma [tw] OR arrhenoblastomas [tw] OR astroblastoma [tw] OR astroblastomas [tw] OR astrocytoma [tw] OR astrocytomas [tw] OR astroglioma [tw] OR astrogliomas [tw] OR atypia [tw] OR baltoma [tw] OR basiloma [tw] OR basilomas [tw] OR biochemotherapies [tw] OR biochemotherapy [tw] OR bioradiotherapy [tw] OR Birt-Hogg-Dube [tw] OR blastoma [tw] OR blastomas [tw] OR Buschke-Lowenstein [tw] OR cachexia [tw] OR cancer [tw] OR cancerous [tw] OR cancers [tw] OR carcinogen [tw] OR carcinogenesis [tw] OR carcinogenic [tw] OR carcinogens [tw] OR carcinoid [tw] OR carcinoma [tw] OR carcinomas [tw] OR carcinomatosis [tw] OR carcinosarcoma [tw] OR carcinosarcomas [tw] OR cavernoma [tw] OR cavernomas [tw] OR cementoma [tw] OR cementomas [tw] OR cerbB2 [tw] OR ceruminoma [tw] OR ceruminomas [tw] OR chemodectoma [tw] OR chemodectomas [tw] OR chemoimmunoradiotherapy [tw] OR chemoimmunotherapies [tw] OR chemoimmunotherapy [tw] OR chemoprevention [tw] OR chemoradiation [tw] OR chemoradiotherapies [tw] OR chemoradiotherapy [tw] OR cherubism [tw] OR chloroma [tw] OR chloromas [tw] OR cholangiocarcinoma [tw] OR cholangiocarcinomas [tw] OR cholangiohepatoma [tw] OR cholangioma [tw] OR cholangiomas [tw] OR cholangiosarcoma [tw] OR cholesteatoma [tw] OR cholesteatomas [tw] OR chondroblastoma [tw] OR chondroblastomas [tw] OR chondroma [tw] OR chondromas [tw] OR chondrosarcoma [tw] OR chondrosarcomas [tw] OR chordoma [tw] OR chordomas [tw] OR chorioadenoma [tw] OR chorioadenomas [tw] OR chorioangioma [tw] OR chorioangiomas [tw] OR choriocarcinoma [tw] OR choriocarcinomas [tw] OR chorioepithelioma [tw] OR chorioepitheliomas [tw] OR chorionepithelioma [tw] OR chorionepitheliomas [tw] OR choristoma [tw] OR choristomas [tw] OR chromaffinoma [tw] OR chromaffinomas [tw] OR cocarcinogenesis [tw] OR collagenoma [tw] OR collagenomas [tw] OR colonoscopies [tw] OR coloscopy [tw] OR coloscopies [tw] OR comedocarcinoma [tw] OR comedocarcinomas [tw] OR condyloma [tw] OR condylomas [tw] OR corticotropinoma [tw] OR corticotropinomas [tw] OR craniopharyngioma [tw] OR craniopharyngiomas [tw] OR cylindroma [tw] OR cylindromas [tw] OR cyst [tw] OR cysts [tw] OR cystadenocarcinoma [tw] OR cystadenocarcinomas [tw] OR cystadenofibroma [tw] OR cystadenofibromas [tw] OR cystadenoma [tw] OR cystadenomas [tw] OR cystoma [tw] OR cystomas [tw] OR cystosarcoma [tw] OR cystosarcomas [tw] OR dentinoma [tw] OR dentinomas [tw] OR dermatofibroma [tw] OR dermatofibromas [tw] OR dermatofibrosarcoma [tw] OR dermatofibrosarcomas [tw] OR dermoid [tw] OR desmoid [tw] OR desmoplastic [tw] OR dictyoma [tw] OR dysgerminoma [tw] OR dysgerminomas [tw] OR dyskeratoma [tw] OR dyskeratomas [tw] OR dysmyelopoiesis [tw] OR dysplasia [tw] OR dysplastic [tw] OR ectomesenchymoma [tw] OR ectomesenchymomas [tw] OR elastofibroma [tw] OR elastofibromas [tw] OR enchondroma [tw] OR enchondromas [tw] OR enchondromatosis [tw] OR endothelioma [tw] OR endotheliomas [tw] OR ependymoblastoma [tw] OR ependymoblastomas [tw] OR ependymoma [tw] OR ependymomas [tw] OR epidermoid [tw] OR epithelioma [tw] OR epitheliomas [tw] OR erythroleukaemia [tw] OR erythroleukaemias [tw] OR erythroleukemia [tw] OR erythroleukemias [tw] OR erythroplakia [tw] OR erythroplakias [tw] OR erythroplasia [tw] OR esthesioneuroblastoma [tw] OR esthesioneuroblastomas [tw] OR esthesioneuroepithelioma [tw] OR esthesioneuroepitheliomas [tw] OR exostosis [tw] OR fibroadenoma [tw] OR fibroadenomas [tw] OR fibroadenosarcoma [tw] OR fibroadenosis [tw] OR fibrochondrosarcoma [tw] OR fibroelastoma [tw] OR fibroelastomas [tw] OR fibroepithelioma [tw] OR fibroepitheliomas [tw] OR fibrofolliculoma [tw] OR fibrofolliculomas [tw] OR fibroid [tw] OR fibroids [tw] OR fibrolipoma [tw] OR fibrolipomas [tw] OR fibroliposarcoma [tw] OR fibroma [tw] OR fibromas [tw] OR fibromatosis [tw] OR fibromyoma [tw] OR fibromyomas [tw] OR fibromyxolipoma [tw] OR fibromyxoma [tw] OR fibromyxomas [tw] OR fibroodontoma [tw] OR fibroodontomas [tw] OR fibrosarcoma [tw] OR fibrosarcomas [tw] OR fibrothecoma [tw] OR fibrothecomas [tw] OR fibroxanthoma [tw] OR fibroxanthomas [tw] OR fibroxanthosarcoma [tw] OR fibroxanthosarcomas [tw] OR ganglioblastoma [tw] OR ganglioblastomas [tw] OR gangliocytoma [tw] OR gangliocytomas [tw] OR ganglioglioma [tw] OR gangliogliomas [tw] OR ganglioneuroblastoma [tw] OR ganglioneuroblastomas [tw] OR ganglioneurofibroma [tw] OR ganglioneurofibromas [tw] OR ganglioneuroma [tw] OR ganglioneuromas [tw] OR gastrinoma [tw] OR gastrinomas [tw] OR germinoma [tw] OR germinomas [tw] OR glioblastoma [tw] OR glioblastomas [tw] OR gliofibroma [tw] OR gliofibromas [tw] OR glioma [tw] OR gliomas [tw] OR gliomatosis [tw] OR glioneuroma [tw] OR glioneuromas [tw] OR gliosarcoma [tw] OR gliosarcomas [tw] OR glomangioma [tw] OR glomangiomas [tw] OR glomangiomatosis [tw] OR glomangiomyoma [tw] OR glomangiomyomas [tw] OR glomangiosarcoma [tw] OR glomangiosarcomas [tw] OR glucagonoma [tw] OR glucagonomas [tw] OR gonadoblastoma [tw] OR gonadoblastomas [tw] OR gonocytoma [tw] OR gonocytomas [tw] OR granuloma [tw] OR granulomas [tw] OR granulomatosis [tw] OR gynaecomastia [tw] OR gynandroblastoma [tw] OR gynecomastia [tw] OR haemangioblastoma [tw] OR haemangioblastomas [tw] OR haemangioma [tw] OR haemangiomas [tw] OR haemangiopericytoma [tw] OR haemangiopericytomas [tw] OR haemangiosarcoma [tw] OR haemangiosarcomas [tw] OR hamartoma [tw] OR hamartomas [tw] OR hemangioblastoma [tw] OR hemangioblastomas [tw] OR hemangioendothelioma [tw] OR hemangioendotheliomas [tw] OR hemangioendotheliosarcoma [tw] OR hemangioendotheliosarcomas [tw] OR hemangioma [tw] OR hemangiomas [tw] OR hemangiomatosis [tw] OR hemangiopericytoma [tw] OR hemangiopericytomas [tw] OR hemangioperithelioma [tw] OR hemangiosarcoma [tw] OR hemangiosarcomas [tw] OR hepatoblastoma [tw] OR hepatoblastomas [tw] OR hepatocarcinoma [tw] OR hepatocarcinomas [tw] OR hepatocholangiocarcinoma [tw] OR hepatocholangiocarcinomas [tw] OR hepatoma [tw] OR hepatomas [tw] OR hibernoma [tw] OR hibernomas [tw] OR hidradenoma [tw] OR hidradenomas [tw] OR hidrocystoma [tw] OR hidrocystomas [tw] OR histiocytoma [tw] OR histiocytomas [tw] OR hodgkin [tw] OR hodgkins [tw] OR hydatidiform [tw] OR hydradenoma [tw] OR hydradenomas [tw] OR hypernephroma [tw] OR hypernephromas [tw] OR immunochemoradiotherapy [tw] OR immunochemotherapies [tw] OR immunochemotherapy [tw] OR immunocytoma [tw] OR immunocytoma [tw] OR immunoradiotherapy [tw] OR insulinomas [tw] OR integrative oncology [tw] OR kasabach-merritt [tw] OR keratoacanthoma [tw] OR keratoacanthomas [tw] OR keratosis [tw] OR leiomyoblastoma [tw] OR leiomyoblastomas [tw] OR leiomyofibroma [tw] OR leiomyofibromas [tw] OR leiomyoma [tw] OR leiomyomas [tw] OR leiomyomatosis [tw] OR leiomyosarcoma [tw] OR leiomyosarcomas [tw] OR leukaemia [tw] OR leukaemias [tw] OR leukemia [tw] OR leukemias [tw] OR leukoplakia [tw] OR leukoplakias [tw] OR lipoadenoma [tw] OR lipoadenomas [tw] OR lipoblastoma [tw] OR lipoblastomas [tw] OR lipoblastomatosis [tw] OR lipoma [tw] OR lipomas [tw] OR lipomatosis [tw] OR liposarcoma [tw] OR liposarcomas [tw] OR luteinoma [tw] OR luteoma [tw] OR luteomas [tw] OR lymphangioendothelioma [tw] OR lymphangioendotheliomas [tw] OR lymphangioleiomyomatosis [tw] OR lymphangioma [tw] OR lymphangiomas [tw] OR lymphangiomatosis [tw] OR lymphangiomyoma [tw] OR lymphangiomyomas [tw] OR lymphangiomyomatosis [tw] OR lymphangiosarcoma [tw] OR lymphangiosarcomas [tw] OR lymphoepithelioma [tw] OR lymphoepitheliomas [tw] OR lymphoma [tw] OR lymphomas [tw] OR lymphoproliferation [tw] OR lymphoproliferations [tw] OR lymphoproliferative [tw] OR lymphoscintigraphic [tw] OR lymphoscintigraphy [tw] OR macroglobulinemia [tw] OR macroglobulinemias [tw] OR macroprolactinoma [tw] OR malignancies [tw] OR malignancy [tw] OR malignant [tw] OR maltoma [tw] OR maltomas [tw] OR mammogram [tw] OR mammograms [tw] OR masculinovoblastoma [tw] OR mastocytoma [tw] OR mastocytomas [tw] OR mastocytosis [tw] OR mcf-7 [tw] OR medulloblastoma [tw] OR medulloblastomas [tw] OR medullocytoma [tw] OR medullocytomas [tw] OR medulloepithelioma [tw] OR medulloepitheliomas [tw] OR medullomyoblastoma [tw] OR medullomyoblastomas [tw] OR melanoacanthoma [tw] OR melanoacanthomas [tw] OR melanoameloblastoma [tw] OR melanocytoma [tw] OR melanocytomas [tw] OR melanoma [tw] OR melanomas [tw] OR melanomatosis [tw] OR meningioblastoma [tw] OR meningioma [tw] OR meningiomas [tw] OR meningiomatosis [tw] OR mesenchymoma [tw] OR mesenchymomas [tw] OR mesonephroma [tw] OR mesonephromas [tw] OR mesothelioma [tw] OR mesotheliomas [tw] OR metaplasia [tw] OR metastases [tw] OR metastasis [tw] OR metastatic [tw] OR microcarcinoma [tw] OR microcarcinomas [tw] OR microglioma [tw] OR microgliomas [tw] OR micrometastases [tw] OR micrometastasis [tw] OR mucositis [tw] OR myelodysplasia [tw] OR myelodysplasias [tw] OR myelodysplastic [tw] OR myelofibrosis [tw] OR myelolipoma [tw] OR myelolipomas [tw] OR myeloma [tw] OR myelomas [tw] OR myelomatosis [tw] OR myeloproliferation [tw] OR myeloproliferations [tw] OR myeloproliferative [tw] OR myelosuppression [tw] OR myoblastoma [tw] OR myoblastomas [tw] OR myoepithelioma [tw] OR myoepitheliomas [tw] OR myofibroblastoma [tw] OR myofibroblastomas [tw] OR myofibroma [tw] OR myofibromas [tw] OR myofibromatosis [tw] OR myofibrosarcoma [tw] OR myofibrosarcomas [tw] OR myolipoma [tw] OR myolipomas [tw] OR myoma [tw] OR myomas [tw] OR myopericytoma [tw] OR myosarcoma [tw] OR myosarcomas [tw] OR myxofibroma [tw] OR myxofibromas [tw] OR myxolipoma [tw] OR myxolipomas [tw] OR myxoliposarcoma [tw] OR myxoma [tw] OR myxomas [tw] OR naevus [tw] OR neoplasia [tw] OR neoplasia [tw] OR neoplasm [tw] OR neoplasms [tw] OR neoplastic [tw] OR nephroblastoma [tw] OR nephroblastomas [tw] OR neurilemmoma [tw] OR neurilemmomas [tw] OR neurilemmomatosis [tw] OR neurilemoma [tw] OR neurilemomas [tw] OR neurinoma [tw] OR neurinomas [tw] OR neuroblastoma [tw] OR neuroblastomas [tw] OR neurocytoma [tw] OR neurocytomas [tw] OR neuroepithelioma [tw] OR neuroepitheliomas [tw] OR neurofibroma [tw] OR neurofibromas [tw] OR neurofibromatosis [tw] OR neurofibrosarcoma [tw] OR neurofibrosarcomas [tw] OR neurolipocytoma [tw] OR neuroma [tw] OR neuromas [tw] OR neuronevus [tw] OR neurothekeoma [tw] OR neurothekeomas [tw] OR nevus [tw] OR nonhodgkin [tw] OR nonhodgkins [tw] OR nonseminoma [tw] OR nonseminomas [tw] OR nonseminomatous [tw] OR odontoameloblastoma [tw] OR odontoma [tw] OR oligoastrocytoma [tw] OR oligoastrocytomas [tw] OR oligodendroglioma [tw] OR oligodendrogliomas [tw] OR oncocytoma [tw] OR oncocytomas [tw] OR oncogen [tw] OR oncogene [tw] OR oncogenes [tw] OR oncogenesis [tw] OR oncogenic [tw] OR oncogens [tw] OR oncologic [tw] OR oncologist [tw] OR oncologists [tw] OR oncology [tw] OR oncoprotein [tw] OR oncoproteins [tw] OR opsoclonus-myoclonus [tw] OR orchioblastoma [tw] OR orchioblastomas [tw] OR osteoblastoma [tw] OR osteoblastomas [tw] OR osteochondroma [tw] OR osteochondromas [tw] OR osteochondrosarcoma [tw] OR osteochondrosarcomas [tw] OR osteoclastoma [tw] OR osteoclastomas [tw] OR osteofibrosarcoma [tw] OR osteoma [tw] OR osteomas [tw] OR osteosarcoma [tw] OR osteosarcomas [tw] OR pancreatoblastoma [tw] OR pancreatoblastomas [tw] OR papilloma [tw] OR papillomas [tw] OR papillomata [tw] OR papillomatosis [tw] OR papillomavirus [tw] OR papillomaviruses [tw] OR parachordoma [tw] OR parachordomas [tw] OR paraganglioma [tw] OR paragangliomas [tw] OR paraneoplastic [tw] OR perineurioma [tw] OR perineuriomas [tw] OR phaeochromocytoma [tw] OR phaeochromocytomas [tw] OR pheochromoblastoma [tw] OR pheochromoblastomas [tw] OR pheochromocytoma [tw] OR pheochromocytomas [tw] OR pilomatricoma [tw] OR pilomatricomas [tw] OR pilomatrixoma [tw] OR pilomatrixomas [tw] OR pinealblastoma [tw] OR pinealoblastoma [tw] OR pinealoblastomas [tw] OR pinealoma [tw] OR pinealomas [tw] OR pineoblastoma [tw] OR pineoblastomas [tw] OR pineocytoma [tw] OR pineocytomas [tw] OR plasmacytoma [tw] OR plasmacytomas [tw] OR pneumoblastoma [tw] OR pneumoblastomas [tw] OR pneumocytoma [tw] OR polyembryoma [tw] OR polyembryomas [tw] OR polyhistioma [tw] OR polyhistiomas [tw] OR polyp [tw] OR polyposis [tw] OR polyps [tw] OR porocarcinoma [tw] OR porocarcinomas [tw] OR poroma [tw] OR poromas [tw] OR precancer [tw] OR precancerous [tw] OR preleukaemia [tw] OR preleukaemias [tw] OR preleukemia [tw] OR preleukemias [tw] OR premalignant [tw] OR preneoplastic [tw] OR prolactinoma [tw] OR prolactinomas [tw] OR protooncogene [tw] OR protooncogenes [tw] OR pseudotumor [tw] OR pseudotumors [tw] OR radiochemotherapy [tw] OR radioimmunotherapies [tw] OR radioimmunotherapy [tw] OR reninoma [tw] OR reninomas [tw] OR reticuloendothelioma [tw] OR reticuloendotheliomas [tw] OR reticulohistiocytoma [tw] OR reticulohistiocytomas [tw] OR reticulosis [tw] OR retinoblastoma [tw] OR retinoblastomas [tw] OR rhabdomyoma [tw] OR rhabdomyomas [tw] OR rhabdomyosarcoma [tw] OR rhabdomyosarcomas [tw] OR rhabdosarcoma [tw] OR rhabdosarcomas [tw] OR sarcoma [tw] OR sarcomas [tw] OR sarcomatosis [tw] OR schwannoma [tw] OR schwannomas [tw] OR schwannomatosis [tw] OR seminoma [tw] OR seminomas [tw] OR seminomatous [tw] OR somatostatinoma [tw] OR somatostatinomas [tw] OR somatotropinoma [tw] OR somatotropinomas [tw] OR spermatocytoma [tw] OR spiradenoma [tw] OR spiradenomas [tw] OR spongioblastoma [tw] OR spongioblastomas [tw] OR steatocystoma [tw] OR steatocystomas [tw] OR subependymoma [tw] OR subependymomas [tw] OR syringadenoma [tw] OR syringadenomas [tw] OR syringocystadenoma [tw] OR syringocystadenomas [tw] OR syringoma [tw] OR syringomas [tw] OR teratocarcinoma [tw] OR teratocarcinomas [tw] OR teratoma [tw] OR teratomas [tw] OR thecoma [tw] OR thecomas [tw] OR thymolipoma [tw] OR thymolipomas [tw] OR thymoma [tw] OR thymomas [tw] OR trichilemmoma [tw] OR trichilemmomas [tw] OR trichoadenoma [tw] OR trichoblastoma [tw] OR trichoblastomas [tw] OR trichodiscoma [tw] OR trichodiscomas [tw] OR trichoepithelioma [tw] OR trichoepitheliomas [tw] OR trichofolliculoma [tw] OR trichofolliculomas [tw] OR tricholemmoma [tw] OR tricholemmomas [tw] OR tumor [tw] OR tumorgenesis [tw] OR tumorgenic [tw] OR tumorigenesis [tw] OR tumorigenic [tw] OR tumorogenesis [tw] OR tumorogenic [tw] OR tumors [tw] OR tumour [tw] OR tumours [tw] OR vipoma [tw] OR vipomas [tw] OR waldenstrom [tw] OR waldenstroms [tw] OR xanthoastrocytoma [tw] OR xanthoastrocytomas [tw] OR xanthofibroma [tw] OR xanthofibromas [tw] OR xanthogranuloma [tw] OR xanthogranulomas [tw] OR xanthoma [tw] OR xanthomas [tw] OR xanthosarcoma [tw] OR xanthosarcomas [tw] OR Acta Oncol [ta] OR Acta Radiol Oncol Radiat Phys Biol [ta] OR Acta Radiol Oncol [ta] OR Adv Cancer Res [ta] OR Adv Immun Cancer Ther [ta] OR Ai Zheng [ta] OR Am J Cancer [ta] OR Am J Clin Oncol [ta] OR Am Soc Clin Oncol Educ Book [ta] OR Anal Cell Pathol [ta] OR Ann Oncol [ta] OR Ann Surg Oncol [ta] OR Anti cancer Drugs [ta] OR Anticancer Agents Med Chem [ta] OR Anticancer Drug Des [ta] OR Anticancer Res [ta] OR Asia Pac J Clin Oncol [ta] OR BMC Cancer [ta] OR Baillieres Clin Oncol [ta] OR Biochim Biophys Acta [ta] OR Blood Cancer J [ta] OR Br J Cancer Suppl [ta] OR Br J Cancer [ta] OR Brain Tumor Pathol [ta] OR Breast Cancer Res Treat [ta] OR Breast Cancer Res [ta] OR Breast Cancer [ta] OR Breast J [ta] OR Bull Assoc Fr Etud Cancer [ta] OR Bull Cancer Radiother [ta] OR Bull Cancer [ta] OR CA Cancer J Clin [ta] OR Can J Oncol [ta] OR Can Oncol Nurs J [ta] OR Cancer Biochem Biophys [ta] OR Cancer Biol Ther [ta] OR Cancer Biomark [ta] OR Cancer Biother Radiopharm [ta] OR Cancer Biother [ta] OR Cancer Bull [ta] OR Cancer Causes Control [ta] OR Cancer Cell Int [ta] OR Cancer Cell [ta] OR Cancer Cells [ta] OR Cancer Chemother Biol Response Modif [ta] OR Cancer Chemother Pharmacol [ta] OR Cancer Chemother Rep 2 [ta] OR Cancer Chemother Rep 3 [ta] OR Cancer Chemother Rep [ta] OR Cancer Clin Trials [ta] OR Cancer Commun [ta] OR "Cancer Commun (Lond)" [ta] OR Cancer Control [ta] OR Cancer Cytol [ta] OR Cancer Cytopathol [ta] OR Cancer Detect Prev Suppl [ta] OR Cancer Detect Prev [ta] OR Cancer Discov [ta] OR Cancer Drug Deliv [ta] OR Cancer Epidemiol Biomarkers Prev [ta] OR Cancer Epidemiol [ta] OR Cancer Gene Ther [ta] OR Cancer Genet [ta] OR Cancer Genet Cytogenet [ta] OR Cancer Genomics Proteomics [ta] OR Cancer Imaging [ta] OR Cancer Immun [ta] OR Cancer Immunol Immunother [ta] OR Cancer Immunol Res [ta] OR Cancer Inform [ta] OR Cancer Invest [ta] OR Cancer J Sci Am [ta] OR Cancer J [ta] OR Cancer Lett [ta] OR Cancer Med [ta] OR Cancer Metastasis Rev [ta] OR Cancer Microenviron [ta] OR Cancer Nurs [ta] OR Cancer Pract [ta] OR Cancer Prev Control [ta] OR Cancer Prev Res Phila [ta] OR Cancer Radiother [ta] OR Cancer Res Treat [ta] OR Cancer Res [ta] OR Cancer Sci [ta] OR Cancer Surv [ta] OR Cancer Treat Rep [ta] OR Cancer Treat Res [ta] OR Cancer Treat Res Commun [ta] OR Cancer Treat Rev [ta] OR Cancer [ta] OR Carcinogenesis [ta] OR Cell Growth Differ [ta] OR Cell Oncol Dordr [ta] OR Chin Clin Oncol [ta] OR Chin J Cancer [ta] OR Chin J Cancer [ta] OR Clin Breast Cancer [ta] OR Clin Cancer Res [ta] OR Clin Colorectal Cancer [ta] OR Clin Exp Metastasis [ta] OR Clin J Oncol Nurs [ta] OR Clin Lymphoma Myeloma Leuk [ta] OR Clin Lymphoma [ta] OR Clin Oncol R Coll Radiol [ta] OR Clin Oncol [ta] OR Clin Transl Oncol [ta] OR CNS Oncol [ta] OR Contemp Oncol [ta] OR Crit Rev Oncog [ta] OR Crit Rev Oncol Hematol [ta] OR Curr Cancer Drug Targets [ta] OR Curr Oncol Rep [ta] OR Curr Oncol [ta] OR Curr Opin Oncol [ta] OR Curr Probl Cancer [ta] OR Curr Treat Options Oncol [ta] OR Dimens Oncol Nurs [ta] OR Drug Resist Updat [ta] OR Eksp Onkol [ta] OR Endocr Relat Cancer [ta] OR Eur J Cancer B Oral Oncol [ta] OR Eur J Cancer Care Engl [ta] OR Eur J Cancer Clin Oncol [ta] OR Eur J Cancer Prev [ta] OR Eur J Cancer [ta] OR Eur J Gynaecol Oncol [ta] OR Eur J Surg Oncol [ta] OR Front Radiat Ther Oncol [ta] OR Future Oncol [ta] OR Gan No Rinsho [ta] OR Gan To Kagaku Ryoho [ta] OR Gastric Cancer [ta] OR Gastrointest Cancer Res [ta] OR Genes Chromosomes Cancer [ta] OR Gulf J Oncolog [ta] OR Gynecol Oncol [ta] OR Head Neck Oncol [ta] OR Hematol Oncol Clin North Am [ta] OR Hematol Oncol Stem Cell Ther [ta] OR Hematol Oncol [ta] OR Hered Cancer Clin Pract [ta] OR Horm Cancer [ta] OR IARC Monogr Eval Carcinog Risk Chem Hum Suppl [ta] OR IARC Monogr Eval Carcinog Risk Chem Hum [ta] OR IARC Monogr Eval Carcinog Risk Chem Man [ta] OR IARC Monogr Eval Carcinog Risks Hum Suppl [ta] OR IARC Monogr Eval Carcinog Risks Hum [ta] OR IARC Sci Publ [ta] OR Important Adv Oncol [ta] OR Indian J Cancer [ta] OR Infect Agent Cancer [ta] OR Innov Oncol Nurs [ta] OR Int Adv Surg Oncol [ta] OR Int J Biol Markers [ta] OR Int J Cancer Suppl [ta] OR Int J Cancer [ta] OR Int J Clin Oncol [ta] OR Int J Gastrointest Cancer [ta] OR Int J Gynecol Cancer [ta] OR Int J Hyperthermia [ta] OR Int J Oncol [ta] OR Int J Radiat Oncol Biol Phys [ta] OR Int J Surg Oncol [ta] OR Integr Cancer Ther [ta] OR Invasion Metastasis [ta] OR Invest New Drugs [ta] OR J Adolesc Young Adult Oncol [ta] OR J Assoc Pediatr Oncol Nurses [ta] OR J Cancer Educ [ta] OR J Cancer Epidemiol Prev [ta] OR J Cancer Res Clin Oncol [ta] OR J Cancer Res [ta] OR J Cancer Surviv [ta] OR J Chemother [ta] OR J Clin Oncol [ta] OR J Community Support Oncol [ta] OR J Dermatol Surg Oncol [ta] OR J Egypt Natl Canc Inst [ta] OR J Environ Pathol Toxicol Oncol [ta] OR J Exp Clin Cancer Res [ta] OR J Exp Ther Oncol [ta] OR J Geriatr Oncol [ta] OR J Gynecol Oncol [ta] OR J Hematol Oncol [ta] OR J Immunother Emphasis Tumor Immunol [ta] OR J Immunother [ta] OR J Mammary Gland Biol Neoplasia [ta] OR J Med Imaging Radiat Oncol [ta] OR J Natl Cancer Inst Monogr [ta] OR J Natl Cancer Inst [ta] OR J Natl Compr Canc Netw [ta] OR J Neurooncol [ta] OR J Oncol Manag [ta] OR J Oncol Pract [ta] OR J Oncol [ta] OR J Pediatr Hematol Oncol [ta] OR J Pediatr Oncol Nurs [ta] OR J Soc Integr Oncol [ta] OR J Support Oncol [ta] OR J Surg Oncol Suppl [ta] OR J Surg Oncol [ta] OR J Thorac Oncol [ta] OR Jaarb Kankeronderz Kankerbestrijd Ned [ta] OR JAMA Oncol [ta] OR JCO Clin Cancer Inform [ta] OR Jpn J Cancer Res [ta] OR Jpn J Clin Oncol [ta] OR Klin Onkol [ta] OR Lancet Oncol [ta] OR Leuk Lymphoma [ta] OR Leuk Res [ta] OR Leukemia [ta] OR Lung Cancer [ta] OR Lutte Cancer [ta] OR Magy Onkol [ta] OR Med Oncol Tumor Pharmacother [ta] OR Med Oncol [ta] OR Med Pediatr Oncol Suppl [ta] OR Med Pediatr Oncol [ta] OR Melanoma Res [ta] OR Mol Cancer Res [ta] OR Mol Cancer Ther [ta] OR Mol Cancer [ta] OR Mol Oncol [ta] OR Monogr Neoplast Dis Var Sites [ta] OR NCI Monogr [ta] OR Nat Rev Cancer [ta] OR Nat Rev Clin Oncol [ta] OR Natl Cancer Inst Monogr [ta] OR Natl Cancer Inst Res Rep [ta] OR Neoplasia [ta] OR Neoplasma [ta] OR Neuro oncol [ta] OR Nippon Gan Chiryo Gakkai Shi [ta] OR Noshuyo Byori [ta] OR Nutr Cancer [ta] OR ONS Connect [ta] OR ONS News [ta] OR Oncogene Res [ta] OR Oncogene [ta] OR Oncol Nurs Forum [ta] OR Oncol Rep [ta] OR Oncol Res [ta] OR Oncol Res Treat [ta] OR Oncologist [ta] OR Oncology Huntingt [ta] OR Oncology [ta] OR Oncotarget [ta] OR Onkologie [ta] OR Open Clin Cancer J [ta] OR Oral Oncol [ta] OR Papillomavirus Res [ta] OR Pathol Oncol Res [ta] OR Pediatr Blood Cancer [ta] OR Pediatr Hematol Oncol [ta] OR Pigment Cell Melanoma Res [ta] OR Pract Radiat Oncol [ta] OR Princess Takamatsu Symp [ta] OR Proc Am Assoc Cancer Res [ta] OR Proc Can Cancer Conf [ta] OR Proc Natl Cancer Conf [ta] OR Prog Clin Cancer [ta] OR Prog Exp Tumor Res [ta] OR Prog Tumor Res [ta] OR Prostate Cancer Prostatic Dis [ta] OR Psychooncology [ta] OR Radiat Oncol Investig [ta] OR Radiat Oncol [ta] OR Radiol Oncol [ta] OR Radiother Oncol [ta] OR Recent Results Cancer Res [ta] OR Rep Carcinog Backgr Doc [ta] OR Rev Mex Cir Ginecol Cancer [ta] OR S Afr Cancer Bull [ta] OR Sci Rep Res Inst Tohoku Univ Med [ta] OR Sel Cancer Ther [ta] OR Semin Cancer Biol [ta] OR Semin Oncol Nurs [ta] OR Semin Oncol [ta] OR Semin Radiat Oncol [ta] OR Semin Surg Oncol [ta] OR Semin Urol Oncol [ta] OR Strahlenther Onkol [ta] OR Suppl J Med Oncol Tumor Pharmacother [ta] OR Suppl Tumori [ta] OR Support Cancer Ther [ta] OR Support Care Cancer [ta] OR Surg Oncol Clin N Am [ta] OR Surg Oncol [ta] OR Symp Fundam Cancer Res [ta] OR Target Oncol [ta] OR Technol Cancer Res Treat [ta] OR Thorac Cancer [ta] OR Transl Oncol [ta] OR Tumor Res [ta] OR Tumori [ta] OR Tumour Biol [ta] OR Urol Oncol [ta] OR Vet Comp Oncol [ta] OR Vopr Onkol [ta] OR World J Surg Oncol [ta] OR Z Krebsforsch Klin Onkol Cancer Res Clin Oncol [ta] OR Z Krebsforsch [ta] OR Zhongguo Fei Ai Za Zhi [ta] OR Zhonghua Zhong Liu Za Zhi [ta])

**Web of Science Core Collection search strategy for cancer**

TS=((neoplasms OR American Cancer Society OR angiogenesis inducing agents OR antibodies, neoplasm OR antigens, neoplasm OR antineoplastic agents OR antineoplastic protocols OR biomarkers, tumor OR biopsy OR biopsy OR bone marrow purging OR bone marrow transplantation OR cancer care facilities OR cancer vaccines OR carcinogenicity tests OR carcinogens OR chemoembolization, therapeutic OR clonal evolution OR clonal evolution OR colonography, computed tomographic OR colonoscopy OR colposcopy OR combined modality therapy OR cryosurgery OR cytapheresis OR dna, neoplasm OR drug resistance, neoplasm OR drug screening assays, antitumor OR early detection of cancer OR gene expression regulation, neoplastic OR genes, neoplasm OR graft vs tumor effect OR hematopoietic stem cell transplantation OR hematopoietic stem cell mobilization OR immunotherapy, adoptive OR leukostasis OR lymph node excision OR lymphocytes, tumor-infiltrating OR mammography OR mastectomy OR medical oncology OR metastasectomy OR mohs surgery OR myelodysplastic-myeloproliferative diseases OR neoplasm grading OR neoplasm proteins OR neoplasm staging OR neoplasm transplantation OR neoplastic processes OR neoplastic stem cells OR oncogene fusion OR oncogenic viruses OR oncology nursing OR oncology service, hospital OR oncolytic viruses OR papanicolaou test OR papillomavirus vaccines OR peripheral blood stem cell transplantation OR polyomavirus OR radiotherapy OR radiotherapy planning, computer assisted OR rna, neoplasm OR second-look surgery OR SEER program OR stem cell transplantation OR transplantation conditioning OR tumor cells, cultured OR tumor escape OR tumor lysis syndrome OR tumor necrosis factors OR receptors, tumor necrosis factor OR tumor necrosis factor receptor-associated peptides AND proteins OR ultrasonography, mammary OR AACR OR AJCC OR (ASCO NOT fungi) OR IARC OR "National Cancer Institute (U.S.)" OR UICC OR aCML OR AGCUS OR AILD OR AML OR ANLL OR ASCUS OR ATLL OR BRCA OR BRCA1 OR BRCA2 OR CIN OR CLL OR CMML OR CMPD OR ECCL OR EGIST OR FMTC OR GLNH OR HNPCC OR HNSCC OR HPV OR HSIL OR ICD O OR JCML OR JMML OR LGLL OR MGUS OR MLH1 OR MPD OR MSH2 OR NSCLC OR RAEB OR RCMD OR SCLC OR VOD OR 5q syndrome OR BCR ABL OR c erbB 2 OR c erbB2 OR carney complex OR cone biopsy OR denys drash OR essential thrombocythemia OR estrogen receptor negative OR estrogen receptor positive OR li fraumeni OR meigs syndrome OR molar pregnancy OR mycosis fungoides OR peutz jeghers OR sentinel lymph node OR sezary syndrome OR struma ovarii OR sturge weber OR zollinger ellison OR (aberrant AND crypt AND foci ) OR ((anti-n-methyl-d-aspartate OR anti-nmda) AND encephalitis ) OR (barrett AND esophagus ) OR (gestational AND trophoblastic ) OR (microsatellite AND instability ) OR (paget AND (breast OR nipple )) OR (polycythemia AND vera ) OR (radiation AND therapy ) OR (WAGR AND syndrome ) OR (pap AND (smear OR smears )) OR cervical smear OR cervical smears OR pap test OR pap tests OR (PSA AND prostate) OR PSA test OR PSA testing OR (prostate AND specific AND antigen ) OR acanthoma OR acanthomas OR acrochordon OR acrochordons OR acrospiroma OR acrospiromas OR adamantinoma OR adamantinomas OR adenoacanthoma OR adenoacanthomas OR adenoameloblastoma OR adenoameloblastomas OR adenocanthoma OR adenocanthomas OR adenocarcinoma OR adenocarcinomas OR adenofibroma OR adenofibromas OR adenolipoma OR adenolipomas OR adenolymphoma OR adenolymphomas OR adenoma OR adenomas OR adenomatosis OR adenomatous OR adenomyoepithelioma OR adenomyoepitheliomas OR adenomyoma OR adenomyomas OR adenosarcoma OR adenosarcomas OR adenosis OR aesthesioneuroblastoma OR aesthesioneuroblastomas OR ameloblastoma OR ameloblastomas OR amyloidoses OR amyloidosis OR anaplasia OR androblastoma OR androblastomas OR angioblastoma OR angioblastomas OR angioendothelioma OR angioendotheliomas OR angioendotheliomatosis OR angiofibroma OR angiofibromas OR angiofibrosarcoma OR angiogenesis factor OR angiokeratoma OR angiokeratomas OR angioleiomyoma OR angioleiomyomas OR angiolipoma OR angiolipomas OR angioma OR angiomas OR angiomatosis OR angiomyolipoma OR angiomyolipomas OR angiomyoma OR angiomyomas OR angiomyxoma OR angiomyxomas OR angioreticuloma OR angioreticulomas OR angiosarcoma OR angiosarcomas OR anticancer OR anticarcinogenesis OR anticarcinogenic OR antimutagenesis OR antineoplastic OR antioncogene OR antioncogenes OR antitumor OR antitumors OR antitumour OR antitumours OR apudoma OR apudomas OR argentaffinoma OR argentaffinomas OR arrhenoblastoma OR arrhenoblastomas OR astroblastoma OR astroblastomas OR astrocytoma OR astrocytomas OR astroglioma OR astrogliomas OR atypia OR baltoma OR basiloma OR basilomas OR biochemotherapies OR biochemotherapy OR bioradiotherapy OR Birt-Hogg-Dube OR blastoma OR blastomas OR Buschke-Lowenstein OR cachexia OR cancer OR cancerous OR cancers OR carcinogen OR carcinogenesis OR carcinogenic OR carcinogens OR carcinoid OR carcinoma OR carcinomas OR carcinomatosis OR carcinosarcoma OR carcinosarcomas OR cavernoma OR cavernomas OR cementoma OR cementomas OR cerbB2 OR ceruminoma OR ceruminomas OR chemodectoma OR chemodectomas OR chemoimmunoradiotherapy OR chemoimmunotherapies OR chemoimmunotherapy OR chemoprevention OR chemoradiation OR chemoradiotherapies OR chemoradiotherapy OR cherubism OR chloroma OR chloromas OR cholangiocarcinoma OR cholangiocarcinomas OR cholangiohepatoma OR cholangioma OR cholangiomas OR cholangiosarcoma OR cholesteatoma OR cholesteatomas OR chondroblastoma OR chondroblastomas OR chondroma OR chondromas OR chondrosarcoma OR chondrosarcomas OR chordoma OR chordomas OR chorioadenoma OR chorioadenomas OR chorioangioma OR chorioangiomas OR choriocarcinoma OR choriocarcinomas OR chorioepithelioma OR chorioepitheliomas OR chorionepithelioma OR chorionepitheliomas OR choristoma OR choristomas OR chromaffinoma OR chromaffinomas OR cocarcinogenesis OR collagenoma OR collagenomas OR colonoscopies OR coloscopy OR coloscopies OR comedocarcinoma OR comedocarcinomas OR condyloma OR condylomas OR corticotropinoma OR corticotropinomas OR craniopharyngioma OR craniopharyngiomas OR cylindroma OR cylindromas OR cyst OR cysts OR cystadenocarcinoma OR cystadenocarcinomas OR cystadenofibroma OR cystadenofibromas OR cystadenoma OR cystadenomas OR cystoma OR cystomas OR cystosarcoma OR cystosarcomas OR dentinoma OR dentinomas OR dermatofibroma OR dermatofibromas OR dermatofibrosarcoma OR dermatofibrosarcomas OR dermoid OR desmoid OR desmoplastic OR dictyoma OR dysgerminoma OR dysgerminomas OR dyskeratoma OR dyskeratomas OR dysmyelopoiesis OR dysplasia OR dysplastic OR ectomesenchymoma OR ectomesenchymomas OR elastofibroma OR elastofibromas OR enchondroma OR enchondromas OR enchondromatosis OR endothelioma OR endotheliomas OR ependymoblastoma OR ependymoblastomas OR ependymoma OR ependymomas OR epidermoid OR epithelioma OR epitheliomas OR erythroleukaemia OR erythroleukaemias OR erythroleukemia OR erythroleukemias OR erythroplakia OR erythroplakias OR erythroplasia OR esthesioneuroblastoma OR esthesioneuroblastomas OR esthesioneuroepithelioma OR esthesioneuroepitheliomas OR exostosis OR fibroadenoma OR fibroadenomas OR fibroadenosarcoma OR fibroadenosis OR fibrochondrosarcoma OR fibroelastoma OR fibroelastomas OR fibroepithelioma OR fibroepitheliomas OR fibrofolliculoma OR fibrofolliculomas OR fibroid OR fibroids OR fibrolipoma OR fibrolipomas OR fibroliposarcoma OR fibroma OR fibromas OR fibromatosis OR fibromyoma OR fibromyomas OR fibromyxolipoma OR fibromyxoma OR fibromyxomas OR fibroodontoma OR fibroodontomas OR fibrosarcoma OR fibrosarcomas OR fibrothecoma OR fibrothecomas OR fibroxanthoma OR fibroxanthomas OR fibroxanthosarcoma OR fibroxanthosarcomas OR ganglioblastoma OR ganglioblastomas OR gangliocytoma OR gangliocytomas OR ganglioglioma OR gangliogliomas OR ganglioneuroblastoma OR ganglioneuroblastomas OR ganglioneurofibroma OR ganglioneurofibromas OR ganglioneuroma OR ganglioneuromas OR gastrinoma OR gastrinomas OR germinoma OR germinomas OR glioblastoma OR glioblastomas OR gliofibroma OR gliofibromas OR glioma OR gliomas OR gliomatosis OR glioneuroma OR glioneuromas OR gliosarcoma OR gliosarcomas OR glomangioma OR glomangiomas OR glomangiomatosis OR glomangiomyoma OR glomangiomyomas OR glomangiosarcoma OR glomangiosarcomas OR glucagonoma OR glucagonomas OR gonadoblastoma OR gonadoblastomas OR gonocytoma OR gonocytomas OR granuloma OR granulomas OR granulomatosis OR gynaecomastia OR gynandroblastoma OR gynecomastia OR haemangioblastoma OR haemangioblastomas OR haemangioma OR haemangiomas OR haemangiopericytoma OR haemangiopericytomas OR haemangiosarcoma OR haemangiosarcomas OR hamartoma OR hamartomas OR hemangioblastoma OR hemangioblastomas OR hemangioendothelioma OR hemangioendotheliomas OR hemangioendotheliosarcoma OR hemangioendotheliosarcomas OR hemangioma OR hemangiomas OR hemangiomatosis OR hemangiopericytoma OR hemangiopericytomas OR hemangioperithelioma OR hemangiosarcoma OR hemangiosarcomas OR hepatoblastoma OR hepatoblastomas OR hepatocarcinoma OR hepatocarcinomas OR hepatocholangiocarcinoma OR hepatocholangiocarcinomas OR hepatoma OR hepatomas OR hibernoma OR hibernomas OR hidradenoma OR hidradenomas OR hidrocystoma OR hidrocystomas OR histiocytoma OR histiocytomas OR hodgkin OR hodgkins OR hydatidiform OR hydradenoma OR hydradenomas OR hypernephroma OR hypernephromas OR immunochemoradiotherapy OR immunochemotherapies OR immunochemotherapy OR immunocytoma OR immunocytoma OR immunoradiotherapy OR insulinomas OR integrative oncology OR kasabach-merritt OR keratoacanthoma OR keratoacanthomas OR keratosis OR leiomyoblastoma OR leiomyoblastomas OR leiomyofibroma OR leiomyofibromas OR leiomyoma OR leiomyomas OR leiomyomatosis OR leiomyosarcoma OR leiomyosarcomas OR leukaemia OR leukaemias OR leukemia OR leukemias OR leukoplakia OR leukoplakias OR lipoadenoma OR lipoadenomas OR lipoblastoma OR lipoblastomas OR lipoblastomatosis OR lipoma OR lipomas OR lipomatosis OR liposarcoma OR liposarcomas OR luteinoma OR luteoma OR luteomas OR lymphangioendothelioma OR lymphangioendotheliomas OR lymphangioleiomyomatosis OR lymphangioma OR lymphangiomas OR lymphangiomatosis OR lymphangiomyoma OR lymphangiomyomas OR lymphangiomyomatosis OR lymphangiosarcoma OR lymphangiosarcomas OR lymphoepithelioma OR lymphoepitheliomas OR lymphoma OR lymphomas OR lymphoproliferation OR lymphoproliferations OR lymphoproliferative OR lymphoscintigraphic OR lymphoscintigraphy OR macroglobulinemia OR macroglobulinemias OR macroprolactinoma OR malignancies OR malignancy OR malignant OR maltoma OR maltomas OR mammogram OR mammograms OR masculinovoblastoma OR mastocytoma OR mastocytomas OR mastocytosis OR mcf-7 OR medulloblastoma OR medulloblastomas OR medullocytoma OR medullocytomas OR medulloepithelioma OR medulloepitheliomas OR medullomyoblastoma OR medullomyoblastomas OR melanoacanthoma OR melanoacanthomas OR melanoameloblastoma OR melanocytoma OR melanocytomas OR melanoma OR melanomas OR melanomatosis OR meningioblastoma OR meningioma OR meningiomas OR meningiomatosis OR mesenchymoma OR mesenchymomas OR mesonephroma OR mesonephromas OR mesothelioma OR mesotheliomas OR metaplasia OR metastases OR metastasis OR metastatic OR microcarcinoma OR microcarcinomas OR microglioma OR microgliomas OR micrometastases OR micrometastasis OR mucositis OR myelodysplasia OR myelodysplasias OR myelodysplastic OR myelofibrosis OR myelolipoma OR myelolipomas OR myeloma OR myelomas OR myelomatosis OR myeloproliferation OR myeloproliferations OR myeloproliferative OR myelosuppression OR myoblastoma OR myoblastomas OR myoepithelioma OR myoepitheliomas OR myofibroblastoma OR myofibroblastomas OR myofibroma OR myofibromas OR myofibromatosis OR myofibrosarcoma OR myofibrosarcomas OR myolipoma OR myolipomas OR myoma OR myomas OR myopericytoma OR myosarcoma OR myosarcomas OR myxofibroma OR myxofibromas OR myxolipoma OR myxolipomas OR myxoliposarcoma OR myxoma OR myxomas OR naevus OR neoplasia OR neoplasia OR neoplasm OR neoplasms OR neoplastic OR nephroblastoma OR nephroblastomas OR neurilemmoma OR neurilemmomas OR neurilemmomatosis OR neurilemoma OR neurilemomas OR neurinoma OR neurinomas OR neuroblastoma OR neuroblastomas OR neurocytoma OR neurocytomas OR neuroepithelioma OR neuroepitheliomas OR neurofibroma OR neurofibromas OR neurofibromatosis OR neurofibrosarcoma OR neurofibrosarcomas OR neurolipocytoma OR neuroma OR neuromas OR neuronevus OR neurothekeoma OR neurothekeomas OR nevus OR nonhodgkin OR nonhodgkins OR nonseminoma OR nonseminomas OR nonseminomatous OR odontoameloblastoma OR odontoma OR oligoastrocytoma OR oligoastrocytomas OR oligodendroglioma OR oligodendrogliomas OR oncocytoma OR oncocytomas OR oncogen OR oncogene OR oncogenes OR oncogenesis OR oncogenic OR oncogens OR oncologic OR oncologist OR oncologists OR oncology OR oncoprotein OR oncoproteins OR opsoclonus-myoclonus OR orchioblastoma OR orchioblastomas OR osteoblastoma OR osteoblastomas OR osteochondroma OR osteochondromas OR osteochondrosarcoma OR osteochondrosarcomas OR osteoclastoma OR osteoclastomas OR osteofibrosarcoma OR osteoma OR osteomas OR osteosarcoma OR osteosarcomas OR pancreatoblastoma OR pancreatoblastomas OR papilloma OR papillomas OR papillomata OR papillomatosis OR papillomavirus OR papillomaviruses OR parachordoma OR parachordomas OR paraganglioma OR paragangliomas OR paraneoplastic OR perineurioma OR perineuriomas OR phaeochromocytoma OR phaeochromocytomas OR pheochromoblastoma OR pheochromoblastomas OR pheochromocytoma OR pheochromocytomas OR pilomatricoma OR pilomatricomas OR pilomatrixoma OR pilomatrixomas OR pinealblastoma OR pinealoblastoma OR pinealoblastomas OR pinealoma OR pinealomas OR pineoblastoma OR pineoblastomas OR pineocytoma OR pineocytomas OR plasmacytoma OR plasmacytomas OR pneumoblastoma OR pneumoblastomas OR pneumocytoma OR polyembryoma OR polyembryomas OR polyhistioma OR polyhistiomas OR polyp OR polyposis OR polyps OR porocarcinoma OR porocarcinomas OR poroma OR poromas OR precancer OR precancerous OR preleukaemia OR preleukaemias OR preleukemia OR preleukemias OR premalignant OR preneoplastic OR prolactinoma OR prolactinomas OR protooncogene OR protooncogenes OR pseudotumor OR pseudotumors OR radiochemotherapy OR radioimmunotherapies OR radioimmunotherapy OR reninoma OR reninomas OR reticuloendothelioma OR reticuloendotheliomas OR reticulohistiocytoma OR reticulohistiocytomas OR reticulosis OR retinoblastoma OR retinoblastomas OR rhabdomyoma OR rhabdomyomas OR rhabdomyosarcoma OR rhabdomyosarcomas OR rhabdosarcoma OR rhabdosarcomas OR sarcoma OR sarcomas OR sarcomatosis OR schwannoma OR schwannomas OR schwannomatosis OR seminoma OR seminomas OR seminomatous OR somatostatinoma OR somatostatinomas OR somatotropinoma OR somatotropinomas OR spermatocytoma OR spiradenoma OR spiradenomas OR spongioblastoma OR spongioblastomas OR steatocystoma OR steatocystomas OR subependymoma OR subependymomas OR syringadenoma OR syringadenomas OR syringocystadenoma OR syringocystadenomas OR syringoma OR syringomas OR teratocarcinoma OR teratocarcinomas OR teratoma OR teratomas OR thecoma OR thecomas OR thymolipoma OR thymolipomas OR thymoma OR thymomas OR trichilemmoma OR trichilemmomas OR trichoadenoma OR trichoblastoma OR trichoblastomas OR trichodiscoma OR trichodiscomas OR trichoepithelioma OR trichoepitheliomas OR trichofolliculoma OR trichofolliculomas OR tricholemmoma OR tricholemmomas OR tumor OR tumorgenesis OR tumorgenic OR tumorigenesis OR tumorigenic OR tumorogenesis OR tumorogenic OR tumors OR tumour OR tumours OR vipoma OR vipomas OR waldenstrom OR waldenstroms OR xanthoastrocytoma OR xanthoastrocytomas OR xanthofibroma OR xanthofibromas OR xanthogranuloma OR xanthogranulomas OR xanthoma OR xanthomas OR xanthosarcoma OR xanthosarcomas OR Acta Oncol OR Acta Radiol Oncol Radiat Phys Biol OR Acta Radiol Oncol OR Adv Cancer Res OR Adv Immun Cancer Ther OR Ai Zheng OR Am J Cancer OR Am J Clin Oncol OR Am Soc Clin Oncol Educ Book OR Anal Cell Pathol OR Ann Oncol OR Ann Surg Oncol OR Anti cancer Drugs OR Anticancer Agents Med Chem OR Anticancer Drug Des OR Anticancer Res OR Asia Pac J Clin Oncol OR BMC Cancer OR Baillieres Clin Oncol OR Biochim Biophys Acta OR Blood Cancer J OR Br J Cancer Suppl OR Br J Cancer OR Brain Tumor Pathol OR Breast Cancer Res Treat OR Breast Cancer Res OR Breast Cancer OR Breast J OR Bull Assoc Fr Etud Cancer OR Bull Cancer Radiother OR Bull Cancer OR CA Cancer J Clin OR Can J Oncol OR Can Oncol Nurs J OR Cancer Biochem Biophys OR Cancer Biol Ther OR Cancer Biomark OR Cancer Biother Radiopharm OR Cancer Biother OR Cancer Bull OR Cancer Causes Control OR Cancer Cell Int OR Cancer Cell OR Cancer Cells OR Cancer Chemother Biol Response Modif OR Cancer Chemother Pharmacol OR Cancer Chemother Rep 2 OR Cancer Chemother Rep 3 OR Cancer Chemother Rep OR Cancer Clin Trials OR Cancer Commun OR "Cancer Commun (Lond)" OR Cancer Control OR Cancer Cytol OR Cancer Cytopathol OR Cancer Detect Prev Suppl OR Cancer Detect Prev OR Cancer Discov OR Cancer Drug Deliv OR Cancer Epidemiol Biomarkers Prev OR Cancer Epidemiol OR Cancer Gene Ther OR Cancer Genet OR Cancer Genet Cytogenet OR Cancer Genomics Proteomics OR Cancer Imaging OR Cancer Immun OR Cancer Immunol Immunother OR Cancer Immunol Res OR Cancer Inform OR Cancer Invest OR Cancer J Sci Am OR Cancer J OR Cancer Lett OR Cancer Med OR Cancer Metastasis Rev OR Cancer Microenviron OR Cancer Nurs OR Cancer Pract OR Cancer Prev Control OR Cancer Prev Res Phila OR Cancer Radiother OR Cancer Res Treat OR Cancer Res OR Cancer Sci OR Cancer Surv OR Cancer Treat Rep OR Cancer Treat Res OR Cancer Treat Res Commun OR Cancer Treat Rev OR Cancer OR Carcinogenesis OR Cell Growth Differ OR Cell Oncol Dordr OR Chin Clin Oncol OR Chin J Cancer OR Chin J Cancer OR Clin Breast Cancer OR Clin Cancer Res OR Clin Colorectal Cancer OR Clin Exp Metastasis OR Clin J Oncol Nurs OR Clin Lymphoma Myeloma Leuk OR Clin Lymphoma OR Clin Oncol R Coll Radiol OR Clin Oncol OR Clin Transl Oncol OR CNS Oncol OR Contemp Oncol OR Crit Rev Oncog OR Crit Rev Oncol Hematol OR Curr Cancer Drug Targets OR Curr Oncol Rep OR Curr Oncol OR Curr Opin Oncol OR Curr Probl Cancer OR Curr Treat Options Oncol OR Dimens Oncol Nurs OR Drug Resist Updat OR Eksp Onkol OR Endocr Relat Cancer OR Eur J Cancer B Oral Oncol OR Eur J Cancer Care Engl OR Eur J Cancer Clin Oncol OR Eur J Cancer Prev OR Eur J Cancer OR Eur J Gynaecol Oncol OR Eur J Surg Oncol OR Front Radiat Ther Oncol OR Future Oncol OR Gan No Rinsho OR Gan To Kagaku Ryoho OR Gastric Cancer OR Gastrointest Cancer Res OR Genes Chromosomes Cancer OR Gulf J Oncolog OR Gynecol Oncol OR Head Neck Oncol OR Hematol Oncol Clin North Am OR Hematol Oncol Stem Cell Ther OR Hematol Oncol OR Hered Cancer Clin Pract OR Horm Cancer OR IARC Monogr Eval Carcinog Risk Chem Hum Suppl OR IARC Monogr Eval Carcinog Risk Chem Hum OR IARC Monogr Eval Carcinog Risk Chem Man OR IARC Monogr Eval Carcinog Risks Hum Suppl OR IARC Monogr Eval Carcinog Risks Hum OR IARC Sci Publ OR Important Adv Oncol OR Indian J Cancer OR Infect Agent Cancer OR Innov Oncol Nurs OR Int Adv Surg Oncol OR Int J Biol Markers OR Int J Cancer Suppl OR Int J Cancer OR Int J Clin Oncol OR Int J Gastrointest Cancer OR Int J Gynecol Cancer OR Int J Hyperthermia OR Int J Oncol OR Int J Radiat Oncol Biol Phys OR Int J Surg Oncol OR Integr Cancer Ther OR Invasion Metastasis OR Invest New Drugs OR J Adolesc Young Adult Oncol OR J Assoc Pediatr Oncol Nurses OR J Cancer Educ OR J Cancer Epidemiol Prev OR J Cancer Res Clin Oncol OR J Cancer Res OR J Cancer Surviv OR J Chemother OR J Clin Oncol OR J Community Support Oncol OR J Dermatol Surg Oncol OR J Egypt Natl Canc Inst OR J Environ Pathol Toxicol Oncol OR J Exp Clin Cancer Res OR J Exp Ther Oncol OR J Geriatr Oncol OR J Gynecol Oncol OR J Hematol Oncol OR J Immunother Emphasis Tumor Immunol OR J Immunother OR J Mammary Gland Biol Neoplasia OR J Med Imaging Radiat Oncol OR J Natl Cancer Inst Monogr OR J Natl Cancer Inst OR J Natl Compr Canc Netw OR J Neurooncol OR J Oncol Manag OR J Oncol Pract OR J Oncol OR J Pediatr Hematol Oncol OR J Pediatr Oncol Nurs OR J Soc Integr Oncol OR J Support Oncol OR J Surg Oncol Suppl OR J Surg Oncol OR J Thorac Oncol OR Jaarb Kankeronderz Kankerbestrijd Ned OR JAMA Oncol OR JCO Clin Cancer Inform OR Jpn J Cancer Res OR Jpn J Clin Oncol OR Klin Onkol OR Lancet Oncol OR Leuk Lymphoma OR Leuk Res OR Leukemia OR Lung Cancer OR Lutte Cancer OR Magy Onkol OR Med Oncol Tumor Pharmacother OR Med Oncol OR Med Pediatr Oncol Suppl OR Med Pediatr Oncol OR Melanoma Res OR Mol Cancer Res OR Mol Cancer Ther OR Mol Cancer OR Mol Oncol OR Monogr Neoplast Dis Var Sites OR NCI Monogr OR Nat Rev Cancer OR Nat Rev Clin Oncol OR Natl Cancer Inst Monogr OR Natl Cancer Inst Res Rep OR Neoplasia OR Neoplasma OR Neuro oncol OR Nippon Gan Chiryo Gakkai Shi OR Noshuyo Byori OR Nutr Cancer OR ONS Connect OR ONS News OR Oncogene Res OR Oncogene OR Oncol Nurs Forum OR Oncol Rep OR Oncol Res OR Oncol Res Treat OR Oncologist OR Oncology Huntingt OR Oncology OR Oncotarget OR Onkologie OR Open Clin Cancer J OR Oral Oncol OR Papillomavirus Res OR Pathol Oncol Res OR Pediatr Blood Cancer OR Pediatr Hematol Oncol OR Pigment Cell Melanoma Res OR Pract Radiat Oncol OR Princess Takamatsu Symp OR Proc Am Assoc Cancer Res OR Proc Can Cancer Conf OR Proc Natl Cancer Conf OR Prog Clin Cancer OR Prog Exp Tumor Res OR Prog Tumor Res OR Prostate Cancer Prostatic Dis OR Psychooncology OR Radiat Oncol Investig OR Radiat Oncol OR Radiol Oncol OR Radiother Oncol OR Recent Results Cancer Res OR Rep Carcinog Backgr Doc OR Rev Mex Cir Ginecol Cancer OR S Afr Cancer Bull OR Sci Rep Res Inst Tohoku Univ Med OR Sel Cancer Ther OR Semin Cancer Biol OR Semin Oncol Nurs OR Semin Oncol OR Semin Radiat Oncol OR Semin Surg Oncol OR Semin Urol Oncol OR Strahlenther Onkol OR Suppl J Med Oncol Tumor Pharmacother OR Suppl Tumori OR Support Cancer Ther OR Support Care Cancer OR Surg Oncol Clin N Am OR Surg Oncol OR Symp Fundam Cancer Res OR Target Oncol OR Technol Cancer Res Treat OR Thorac Cancer OR Transl Oncol OR Tumor Res OR Tumori OR Tumour Biol OR Urol Oncol OR Vet Comp Oncol OR Vopr Onkol OR World J Surg Oncol OR Z Krebsforsch Klin Onkol Cancer Res Clin Oncol OR Z Krebsforsch OR Zhongguo Fei Ai Za Zhi OR Zhonghua Zhong Liu Za Zhi ))

**Embase search strategy for cancer**

(neoplasms OR American Cancer Society OR angiogenesis inducing agents OR antibodies, neoplasm OR antigens, neoplasm OR antineoplastic agents OR antineoplastic protocols OR biomarkers, tumor OR biopsy OR biopsy OR bone marrow purging OR bone marrow transplantation OR cancer care facilities OR cancer vaccines OR carcinogenicity tests OR carcinogens OR chemoembolization, therapeutic OR clonal evolution OR clonal evolution OR colonography, computed tomographic OR colonoscopy OR colposcopy OR combined modality therapy OR cryosurgery OR cytapheresis OR dna, neoplasm OR drug resistance, neoplasm OR drug screening assays, antitumor OR early detection of cancer OR gene expression regulation, neoplastic OR genes, neoplasm OR graft vs tumor effect OR hematopoietic stem cell transplantation OR hematopoietic stem cell mobilization OR immunotherapy, adoptive OR leukostasis OR lymph node excision OR lymphocytes, tumor-infiltrating OR mammography OR mastectomy OR medical oncology OR metastasectomy OR mohs surgery OR myelodysplastic-myeloproliferative diseases OR neoplasm grading OR neoplasm proteins OR neoplasm staging OR neoplasm transplantation OR neoplastic processes OR neoplastic stem cells OR oncogene fusion OR oncogenic viruses OR oncology nursing OR oncology service, hospital OR oncolytic viruses OR papanicolaou test OR papillomavirus vaccines OR peripheral blood stem cell transplantation OR polyomavirus OR radiotherapy OR radiotherapy planning, computer assisted OR rna, neoplasm OR second-look surgery OR SEER program OR stem cell transplantation OR transplantation conditioning OR tumor cells, cultured OR tumor escape OR tumor lysis syndrome OR tumor necrosis factors OR receptors, tumor necrosis factor OR tumor necrosis factor receptor-associated peptides AND proteins OR ultrasonography, mammary OR AACR OR AJCC OR (ASCO NOT fungi) OR IARC OR "National Cancer Institute (U.S.)" OR UICC OR aCML OR AGCUS OR AILD OR AML OR ANLL OR ASCUS OR ATLL OR BRCA OR BRCA1 OR BRCA2 OR CIN OR CLL OR CMML OR CMPD OR ECCL OR EGIST OR FMTC OR GLNH OR HNPCC OR HNSCC OR HPV OR HSIL OR ICD O OR JCML OR JMML OR LGLL OR MGUS OR MLH1 OR MPD OR MSH2 OR NSCLC OR RAEB OR RCMD OR SCLC OR VOD OR 5q syndrome OR BCR ABL OR c erbB 2 OR c erbB2 OR carney complex OR cone biopsy OR denys drash OR essential thrombocythemia OR estrogen receptor negative OR estrogen receptor positive OR li fraumeni OR meigs syndrome OR molar pregnancy OR mycosis fungoides OR peutz jeghers OR sentinel lymph node OR sezary syndrome OR struma ovarii OR sturge weber OR zollinger ellison OR (aberrant AND crypt AND foci ) OR ((anti-n-methyl-d-aspartate OR anti-nmda) AND encephalitis ) OR (barrett AND esophagus ) OR (gestational AND trophoblastic ) OR (microsatellite AND instability ) OR (paget AND (breast OR nipple )) OR (polycythemia AND vera ) OR (radiation AND therapy ) OR (WAGR AND syndrome ) OR (pap AND (smear OR smears )) OR cervical smear OR cervical smears OR pap test OR pap tests OR (PSA AND prostate) OR PSA test OR PSA testing OR (prostate AND specific AND antigen ) OR acanthoma OR acanthomas OR acrochordon OR acrochordons OR acrospiroma OR acrospiromas OR adamantinoma OR adamantinomas OR adenoacanthoma OR adenoacanthomas OR adenoameloblastoma OR adenoameloblastomas OR adenocanthoma OR adenocanthomas OR adenocarcinoma OR adenocarcinomas OR adenofibroma OR adenofibromas OR adenolipoma OR adenolipomas OR adenolymphoma OR adenolymphomas OR adenoma OR adenomas OR adenomatosis OR adenomatous OR adenomyoepithelioma OR adenomyoepitheliomas OR adenomyoma OR adenomyomas OR adenosarcoma OR adenosarcomas OR adenosis OR aesthesioneuroblastoma OR aesthesioneuroblastomas OR ameloblastoma OR ameloblastomas OR amyloidoses OR amyloidosis OR anaplasia OR androblastoma OR androblastomas OR angioblastoma OR angioblastomas OR angioendothelioma OR angioendotheliomas OR angioendotheliomatosis OR angiofibroma OR angiofibromas OR angiofibrosarcoma OR angiogenesis factor OR angiokeratoma OR angiokeratomas OR angioleiomyoma OR angioleiomyomas OR angiolipoma OR angiolipomas OR angioma OR angiomas OR angiomatosis OR angiomyolipoma OR angiomyolipomas OR angiomyoma OR angiomyomas OR angiomyxoma OR angiomyxomas OR angioreticuloma OR angioreticulomas OR angiosarcoma OR angiosarcomas OR anticancer OR anticarcinogenesis OR anticarcinogenic OR antimutagenesis OR antineoplastic OR antioncogene OR antioncogenes OR antitumor OR antitumors OR antitumour OR antitumours OR apudoma OR apudomas OR argentaffinoma OR argentaffinomas OR arrhenoblastoma OR arrhenoblastomas OR astroblastoma OR astroblastomas OR astrocytoma OR astrocytomas OR astroglioma OR astrogliomas OR atypia OR baltoma OR basiloma OR basilomas OR biochemotherapies OR biochemotherapy OR bioradiotherapy OR Birt-Hogg-Dube OR blastoma OR blastomas OR Buschke-Lowenstein OR cachexia OR cancer OR cancerous OR cancers OR carcinogen OR carcinogenesis OR carcinogenic OR carcinogens OR carcinoid OR carcinoma OR carcinomas OR carcinomatosis OR carcinosarcoma OR carcinosarcomas OR cavernoma OR cavernomas OR cementoma OR cementomas OR cerbB2 OR ceruminoma OR ceruminomas OR chemodectoma OR chemodectomas OR chemoimmunoradiotherapy OR chemoimmunotherapies OR chemoimmunotherapy OR chemoprevention OR chemoradiation OR chemoradiotherapies OR chemoradiotherapy OR cherubism OR chloroma OR chloromas OR cholangiocarcinoma OR cholangiocarcinomas OR cholangiohepatoma OR cholangioma OR cholangiomas OR cholangiosarcoma OR cholesteatoma OR cholesteatomas OR chondroblastoma OR chondroblastomas OR chondroma OR chondromas OR chondrosarcoma OR chondrosarcomas OR chordoma OR chordomas OR chorioadenoma OR chorioadenomas OR chorioangioma OR chorioangiomas OR choriocarcinoma OR choriocarcinomas OR chorioepithelioma OR chorioepitheliomas OR chorionepithelioma OR chorionepitheliomas OR choristoma OR choristomas OR chromaffinoma OR chromaffinomas OR cocarcinogenesis OR collagenoma OR collagenomas OR colonoscopies OR coloscopy OR coloscopies OR comedocarcinoma OR comedocarcinomas OR condyloma OR condylomas OR corticotropinoma OR corticotropinomas OR craniopharyngioma OR craniopharyngiomas OR cylindroma OR cylindromas OR cyst OR cysts OR cystadenocarcinoma OR cystadenocarcinomas OR cystadenofibroma OR cystadenofibromas OR cystadenoma OR cystadenomas OR cystoma OR cystomas OR cystosarcoma OR cystosarcomas OR dentinoma OR dentinomas OR dermatofibroma OR dermatofibromas OR dermatofibrosarcoma OR dermatofibrosarcomas OR dermoid OR desmoid OR desmoplastic OR dictyoma OR dysgerminoma OR dysgerminomas OR dyskeratoma OR dyskeratomas OR dysmyelopoiesis OR dysplasia OR dysplastic OR ectomesenchymoma OR ectomesenchymomas OR elastofibroma OR elastofibromas OR enchondroma OR enchondromas OR enchondromatosis OR endothelioma OR endotheliomas OR ependymoblastoma OR ependymoblastomas OR ependymoma OR ependymomas OR epidermoid OR epithelioma OR epitheliomas OR erythroleukaemia OR erythroleukaemias OR erythroleukemia OR erythroleukemias OR erythroplakia OR erythroplakias OR erythroplasia OR esthesioneuroblastoma OR esthesioneuroblastomas OR esthesioneuroepithelioma OR esthesioneuroepitheliomas OR exostosis OR fibroadenoma OR fibroadenomas OR fibroadenosarcoma OR fibroadenosis OR fibrochondrosarcoma OR fibroelastoma OR fibroelastomas OR fibroepithelioma OR fibroepitheliomas OR fibrofolliculoma OR fibrofolliculomas OR fibroid OR fibroids OR fibrolipoma OR fibrolipomas OR fibroliposarcoma OR fibroma OR fibromas OR fibromatosis OR fibromyoma OR fibromyomas OR fibromyxolipoma OR fibromyxoma OR fibromyxomas OR fibroodontoma OR fibroodontomas OR fibrosarcoma OR fibrosarcomas OR fibrothecoma OR fibrothecomas OR fibroxanthoma OR fibroxanthomas OR fibroxanthosarcoma OR fibroxanthosarcomas OR ganglioblastoma OR ganglioblastomas OR gangliocytoma OR gangliocytomas OR ganglioglioma OR gangliogliomas OR ganglioneuroblastoma OR ganglioneuroblastomas OR ganglioneurofibroma OR ganglioneurofibromas OR ganglioneuroma OR ganglioneuromas OR gastrinoma OR gastrinomas OR germinoma OR germinomas OR glioblastoma OR glioblastomas OR gliofibroma OR gliofibromas OR glioma OR gliomas OR gliomatosis OR glioneuroma OR glioneuromas OR gliosarcoma OR gliosarcomas OR glomangioma OR glomangiomas OR glomangiomatosis OR glomangiomyoma OR glomangiomyomas OR glomangiosarcoma OR glomangiosarcomas OR glucagonoma OR glucagonomas OR gonadoblastoma OR gonadoblastomas OR gonocytoma OR gonocytomas OR granuloma OR granulomas OR granulomatosis OR gynaecomastia OR gynandroblastoma OR gynecomastia OR haemangioblastoma OR haemangioblastomas OR haemangioma OR haemangiomas OR haemangiopericytoma OR haemangiopericytomas OR haemangiosarcoma OR haemangiosarcomas OR hamartoma OR hamartomas OR hemangioblastoma OR hemangioblastomas OR hemangioendothelioma OR hemangioendotheliomas OR hemangioendotheliosarcoma OR hemangioendotheliosarcomas OR hemangioma OR hemangiomas OR hemangiomatosis OR hemangiopericytoma OR hemangiopericytomas OR hemangioperithelioma OR hemangiosarcoma OR hemangiosarcomas OR hepatoblastoma OR hepatoblastomas OR hepatocarcinoma OR hepatocarcinomas OR hepatocholangiocarcinoma OR hepatocholangiocarcinomas OR hepatoma OR hepatomas OR hibernoma OR hibernomas OR hidradenoma OR hidradenomas OR hidrocystoma OR hidrocystomas OR histiocytoma OR histiocytomas OR hodgkin OR hodgkins OR hydatidiform OR hydradenoma OR hydradenomas OR hypernephroma OR hypernephromas OR immunochemoradiotherapy OR immunochemotherapies OR immunochemotherapy OR immunocytoma OR immunocytoma OR immunoradiotherapy OR insulinomas OR integrative oncology OR kasabach-merritt OR keratoacanthoma OR keratoacanthomas OR keratosis OR leiomyoblastoma OR leiomyoblastomas OR leiomyofibroma OR leiomyofibromas OR leiomyoma OR leiomyomas OR leiomyomatosis OR leiomyosarcoma OR leiomyosarcomas OR leukaemia OR leukaemias OR leukemia OR leukemias OR leukoplakia OR leukoplakias OR lipoadenoma OR lipoadenomas OR lipoblastoma OR lipoblastomas OR lipoblastomatosis OR lipoma OR lipomas OR lipomatosis OR liposarcoma OR liposarcomas OR luteinoma OR luteoma OR luteomas OR lymphangioendothelioma OR lymphangioendotheliomas OR lymphangioleiomyomatosis OR lymphangioma OR lymphangiomas OR lymphangiomatosis OR lymphangiomyoma OR lymphangiomyomas OR lymphangiomyomatosis OR lymphangiosarcoma OR lymphangiosarcomas OR lymphoepithelioma OR lymphoepitheliomas OR lymphoma OR lymphomas OR lymphoproliferation OR lymphoproliferations OR lymphoproliferative OR lymphoscintigraphic OR lymphoscintigraphy OR macroglobulinemia OR macroglobulinemias OR macroprolactinoma OR malignancies OR malignancy OR malignant OR maltoma OR maltomas OR mammogram OR mammograms OR masculinovoblastoma OR mastocytoma OR mastocytomas OR mastocytosis OR mcf-7 OR medulloblastoma OR medulloblastomas OR medullocytoma OR medullocytomas OR medulloepithelioma OR medulloepitheliomas OR medullomyoblastoma OR medullomyoblastomas OR melanoacanthoma OR melanoacanthomas OR melanoameloblastoma OR melanocytoma OR melanocytomas OR melanoma OR melanomas OR melanomatosis OR meningioblastoma OR meningioma OR meningiomas OR meningiomatosis OR mesenchymoma OR mesenchymomas OR mesonephroma OR mesonephromas OR mesothelioma OR mesotheliomas OR metaplasia OR metastases OR metastasis OR metastatic OR microcarcinoma OR microcarcinomas OR microglioma OR microgliomas OR micrometastases OR micrometastasis OR mucositis OR myelodysplasia OR myelodysplasias OR myelodysplastic OR myelofibrosis OR myelolipoma OR myelolipomas OR myeloma OR myelomas OR myelomatosis OR myeloproliferation OR myeloproliferations OR myeloproliferative OR myelosuppression OR myoblastoma OR myoblastomas OR myoepithelioma OR myoepitheliomas OR myofibroblastoma OR myofibroblastomas OR myofibroma OR myofibromas OR myofibromatosis OR myofibrosarcoma OR myofibrosarcomas OR myolipoma OR myolipomas OR myoma OR myomas OR myopericytoma OR myosarcoma OR myosarcomas OR myxofibroma OR myxofibromas OR myxolipoma OR myxolipomas OR myxoliposarcoma OR myxoma OR myxomas OR naevus OR neoplasia OR neoplasia OR neoplasm OR neoplasms OR neoplastic OR nephroblastoma OR nephroblastomas OR neurilemmoma OR neurilemmomas OR neurilemmomatosis OR neurilemoma OR neurilemomas OR neurinoma OR neurinomas OR neuroblastoma OR neuroblastomas OR neurocytoma OR neurocytomas OR neuroepithelioma OR neuroepitheliomas OR neurofibroma OR neurofibromas OR neurofibromatosis OR neurofibrosarcoma OR neurofibrosarcomas OR neurolipocytoma OR neuroma OR neuromas OR neuronevus OR neurothekeoma OR neurothekeomas OR nevus OR nonhodgkin OR nonhodgkins OR nonseminoma OR nonseminomas OR nonseminomatous OR odontoameloblastoma OR odontoma OR oligoastrocytoma OR oligoastrocytomas OR oligodendroglioma OR oligodendrogliomas OR oncocytoma OR oncocytomas OR oncogen OR oncogene OR oncogenes OR oncogenesis OR oncogenic OR oncogens OR oncologic OR oncologist OR oncologists OR oncology OR oncoprotein OR oncoproteins OR opsoclonus-myoclonus OR orchioblastoma OR orchioblastomas OR osteoblastoma OR osteoblastomas OR osteochondroma OR osteochondromas OR osteochondrosarcoma OR osteochondrosarcomas OR osteoclastoma OR osteoclastomas OR osteofibrosarcoma OR osteoma OR osteomas OR osteosarcoma OR osteosarcomas OR pancreatoblastoma OR pancreatoblastomas OR papilloma OR papillomas OR papillomata OR papillomatosis OR papillomavirus OR papillomaviruses OR parachordoma OR parachordomas OR paraganglioma OR paragangliomas OR paraneoplastic OR perineurioma OR perineuriomas OR phaeochromocytoma OR phaeochromocytomas OR pheochromoblastoma OR pheochromoblastomas OR pheochromocytoma OR pheochromocytomas OR pilomatricoma OR pilomatricomas OR pilomatrixoma OR pilomatrixomas OR pinealblastoma OR pinealoblastoma OR pinealoblastomas OR pinealoma OR pinealomas OR pineoblastoma OR pineoblastomas OR pineocytoma OR pineocytomas OR plasmacytoma OR plasmacytomas OR pneumoblastoma OR pneumoblastomas OR pneumocytoma OR polyembryoma OR polyembryomas OR polyhistioma OR polyhistiomas OR polyp OR polyposis OR polyps OR porocarcinoma OR porocarcinomas OR poroma OR poromas OR precancer OR precancerous OR preleukaemia OR preleukaemias OR preleukemia OR preleukemias OR premalignant OR preneoplastic OR prolactinoma OR prolactinomas OR protooncogene OR protooncogenes OR pseudotumor OR pseudotumors OR radiochemotherapy OR radioimmunotherapies OR radioimmunotherapy OR reninoma OR reninomas OR reticuloendothelioma OR reticuloendotheliomas OR reticulohistiocytoma OR reticulohistiocytomas OR reticulosis OR retinoblastoma OR retinoblastomas OR rhabdomyoma OR rhabdomyomas OR rhabdomyosarcoma OR rhabdomyosarcomas OR rhabdosarcoma OR rhabdosarcomas OR sarcoma OR sarcomas OR sarcomatosis OR schwannoma OR schwannomas OR schwannomatosis OR seminoma OR seminomas OR seminomatous OR somatostatinoma OR somatostatinomas OR somatotropinoma OR somatotropinomas OR spermatocytoma OR spiradenoma OR spiradenomas OR spongioblastoma OR spongioblastomas OR steatocystoma OR steatocystomas OR subependymoma OR subependymomas OR syringadenoma OR syringadenomas OR syringocystadenoma OR syringocystadenomas OR syringoma OR syringomas OR teratocarcinoma OR teratocarcinomas OR teratoma OR teratomas OR thecoma OR thecomas OR thymolipoma OR thymolipomas OR thymoma OR thymomas OR trichilemmoma OR trichilemmomas OR trichoadenoma OR trichoblastoma OR trichoblastomas OR trichodiscoma OR trichodiscomas OR trichoepithelioma OR trichoepitheliomas OR trichofolliculoma OR trichofolliculomas OR tricholemmoma OR tricholemmomas OR tumor OR tumorgenesis OR tumorgenic OR tumorigenesis OR tumorigenic OR tumorogenesis OR tumorogenic OR tumors OR tumour OR tumours OR vipoma OR vipomas OR waldenstrom OR waldenstroms OR xanthoastrocytoma OR xanthoastrocytomas OR xanthofibroma OR xanthofibromas OR xanthogranuloma OR xanthogranulomas OR xanthoma OR xanthomas OR xanthosarcoma OR xanthosarcomas OR Acta Oncol OR Acta Radiol Oncol Radiat Phys Biol OR Acta Radiol Oncol OR Adv Cancer Res OR Adv Immun Cancer Ther OR Ai Zheng OR Am J Cancer OR Am J Clin Oncol OR Am Soc Clin Oncol Educ Book OR Anal Cell Pathol OR Ann Oncol OR Ann Surg Oncol OR Anti cancer Drugs OR Anticancer Agents Med Chem OR Anticancer Drug Des OR Anticancer Res OR Asia Pac J Clin Oncol OR BMC Cancer OR Baillieres Clin Oncol OR Biochim Biophys Acta OR Blood Cancer J OR Br J Cancer Suppl OR Br J Cancer OR Brain Tumor Pathol OR Breast Cancer Res Treat OR Breast Cancer Res OR Breast Cancer OR Breast J OR Bull Assoc Fr Etud Cancer OR Bull Cancer Radiother OR Bull Cancer OR CA Cancer J Clin OR Can J Oncol OR Can Oncol Nurs J OR Cancer Biochem Biophys OR Cancer Biol Ther OR Cancer Biomark OR Cancer Biother Radiopharm OR Cancer Biother OR Cancer Bull OR Cancer Causes Control OR Cancer Cell Int OR Cancer Cell OR Cancer Cells OR Cancer Chemother Biol Response Modif OR Cancer Chemother Pharmacol OR Cancer Chemother Rep 2 OR Cancer Chemother Rep 3 OR Cancer Chemother Rep OR Cancer Clin Trials OR Cancer Commun OR "Cancer Commun (Lond)" OR Cancer Control OR Cancer Cytol OR Cancer Cytopathol OR Cancer Detect Prev Suppl OR Cancer Detect Prev OR Cancer Discov OR Cancer Drug Deliv OR Cancer Epidemiol Biomarkers Prev OR Cancer Epidemiol OR Cancer Gene Ther OR Cancer Genet OR Cancer Genet Cytogenet OR Cancer Genomics Proteomics OR Cancer Imaging OR Cancer Immun OR Cancer Immunol Immunother OR Cancer Immunol Res OR Cancer Inform OR Cancer Invest OR Cancer J Sci Am OR Cancer J OR Cancer Lett OR Cancer Med OR Cancer Metastasis Rev OR Cancer Microenviron OR Cancer Nurs OR Cancer Pract OR Cancer Prev Control OR Cancer Prev Res Phila OR Cancer Radiother OR Cancer Res Treat OR Cancer Res OR Cancer Sci OR Cancer Surv OR Cancer Treat Rep OR Cancer Treat Res OR Cancer Treat Res Commun OR Cancer Treat Rev OR Cancer OR Carcinogenesis OR Cell Growth Differ OR Cell Oncol Dordr OR Chin Clin Oncol OR Chin J Cancer OR Chin J Cancer OR Clin Breast Cancer OR Clin Cancer Res OR Clin Colorectal Cancer OR Clin Exp Metastasis OR Clin J Oncol Nurs OR Clin Lymphoma Myeloma Leuk OR Clin Lymphoma OR Clin Oncol R Coll Radiol OR Clin Oncol OR Clin Transl Oncol OR CNS Oncol OR Contemp Oncol OR Crit Rev Oncog OR Crit Rev Oncol Hematol OR Curr Cancer Drug Targets OR Curr Oncol Rep OR Curr Oncol OR Curr Opin Oncol OR Curr Probl Cancer OR Curr Treat Options Oncol OR Dimens Oncol Nurs OR Drug Resist Updat OR Eksp Onkol OR Endocr Relat Cancer OR Eur J Cancer B Oral Oncol OR Eur J Cancer Care Engl OR Eur J Cancer Clin Oncol OR Eur J Cancer Prev OR Eur J Cancer OR Eur J Gynaecol Oncol OR Eur J Surg Oncol OR Front Radiat Ther Oncol OR Future Oncol OR Gan No Rinsho OR Gan To Kagaku Ryoho OR Gastric Cancer OR Gastrointest Cancer Res OR Genes Chromosomes Cancer OR Gulf J Oncolog OR Gynecol Oncol OR Head Neck Oncol OR Hematol Oncol Clin North Am OR Hematol Oncol Stem Cell Ther OR Hematol Oncol OR Hered Cancer Clin Pract OR Horm Cancer OR IARC Monogr Eval Carcinog Risk Chem Hum Suppl OR IARC Monogr Eval Carcinog Risk Chem Hum OR IARC Monogr Eval Carcinog Risk Chem Man OR IARC Monogr Eval Carcinog Risks Hum Suppl OR IARC Monogr Eval Carcinog Risks Hum OR IARC Sci Publ OR Important Adv Oncol OR Indian J Cancer OR Infect Agent Cancer OR Innov Oncol Nurs OR Int Adv Surg Oncol OR Int J Biol Markers OR Int J Cancer Suppl OR Int J Cancer OR Int J Clin Oncol OR Int J Gastrointest Cancer OR Int J Gynecol Cancer OR Int J Hyperthermia OR Int J Oncol OR Int J Radiat Oncol Biol Phys OR Int J Surg Oncol OR Integr Cancer Ther OR Invasion Metastasis OR Invest New Drugs OR J Adolesc Young Adult Oncol OR J Assoc Pediatr Oncol Nurses OR J Cancer Educ OR J Cancer Epidemiol Prev OR J Cancer Res Clin Oncol OR J Cancer Res OR J Cancer Surviv OR J Chemother OR J Clin Oncol OR J Community Support Oncol OR J Dermatol Surg Oncol OR J Egypt Natl Canc Inst OR J Environ Pathol Toxicol Oncol OR J Exp Clin Cancer Res OR J Exp Ther Oncol OR J Geriatr Oncol OR J Gynecol Oncol OR J Hematol Oncol OR J Immunother Emphasis Tumor Immunol OR J Immunother OR J Mammary Gland Biol Neoplasia OR J Med Imaging Radiat Oncol OR J Natl Cancer Inst Monogr OR J Natl Cancer Inst OR J Natl Compr Canc Netw OR J Neurooncol OR J Oncol Manag OR J Oncol Pract OR J Oncol OR J Pediatr Hematol Oncol OR J Pediatr Oncol Nurs OR J Soc Integr Oncol OR J Support Oncol OR J Surg Oncol Suppl OR J Surg Oncol OR J Thorac Oncol OR Jaarb Kankeronderz Kankerbestrijd Ned OR JAMA Oncol OR JCO Clin Cancer Inform OR Jpn J Cancer Res OR Jpn J Clin Oncol OR Klin Onkol OR Lancet Oncol OR Leuk Lymphoma OR Leuk Res OR Leukemia OR Lung Cancer OR Lutte Cancer OR Magy Onkol OR Med Oncol Tumor Pharmacother OR Med Oncol OR Med Pediatr Oncol Suppl OR Med Pediatr Oncol OR Melanoma Res OR Mol Cancer Res OR Mol Cancer Ther OR Mol Cancer OR Mol Oncol OR Monogr Neoplast Dis Var Sites OR NCI Monogr OR Nat Rev Cancer OR Nat Rev Clin Oncol OR Natl Cancer Inst Monogr OR Natl Cancer Inst Res Rep OR Neoplasia OR Neoplasma OR Neuro oncol OR Nippon Gan Chiryo Gakkai Shi OR Noshuyo Byori OR Nutr Cancer OR ONS Connect OR ONS News OR Oncogene Res OR Oncogene OR Oncol Nurs Forum OR Oncol Rep OR Oncol Res OR Oncol Res Treat OR Oncologist OR Oncology Huntingt OR Oncology OR Oncotarget OR Onkologie OR Open Clin Cancer J OR Oral Oncol OR Papillomavirus Res OR Pathol Oncol Res OR Pediatr Blood Cancer OR Pediatr Hematol Oncol OR Pigment Cell Melanoma Res OR Pract Radiat Oncol OR Princess Takamatsu Symp OR Proc Am Assoc Cancer Res OR Proc Can Cancer Conf OR Proc Natl Cancer Conf OR Prog Clin Cancer OR Prog Exp Tumor Res OR Prog Tumor Res OR Prostate Cancer Prostatic Dis OR Psychooncology OR Radiat Oncol Investig OR Radiat Oncol OR Radiol Oncol OR Radiother Oncol OR Recent Results Cancer Res OR Rep Carcinog Backgr Doc OR Rev Mex Cir Ginecol Cancer OR S Afr Cancer Bull OR Sci Rep Res Inst Tohoku Univ Med OR Sel Cancer Ther OR Semin Cancer Biol OR Semin Oncol Nurs OR Semin Oncol OR Semin Radiat Oncol OR Semin Surg Oncol OR Semin Urol Oncol OR Strahlenther Onkol OR Suppl J Med Oncol Tumor Pharmacother OR Suppl Tumori OR Support Cancer Ther OR Support Care Cancer OR Surg Oncol Clin N Am OR Surg Oncol OR Symp Fundam Cancer Res OR Target Oncol OR Technol Cancer Res Treat OR Thorac Cancer OR Transl Oncol OR Tumor Res OR Tumori OR Tumour Biol OR Urol Oncol OR Vet Comp Oncol OR Vopr Onkol OR World J Surg Oncol OR Z Krebsforsch Klin Onkol Cancer Res Clin Oncol OR Z Krebsforsch OR Zhongguo Fei Ai Za Zhi OR Zhonghua Zhong Liu Za Zhi )
